# IonFusion: Multimodal Fusion for Hierarchical Classification and Functional Annotation of Ion Channels and Transporters

**DOI:** 10.3390/molecules31203679

**Published:** 2026-10-14

**Authors:** Ruiqing Zheng, Wentao Yu, Qiurong Yang, Zelin Zhao, Wenxuan He, Shubiao Qu, Taozhe Li, Wenkang Wang

**Affiliations:** School of Computer Science and Engineering, Central South University, Changsha 410083, China; zqr@csu.edu.cn (R.Z.); 254711028@csu.edu.cn (W.Y.); yangqr@csu.edu.cn (Q.Y.); zhaozelin@csu.edu.cn (Z.Z.); hewenxuan@csu.edu.cn (W.H.); shubiaoqu@163.com (S.Q.); 254712116@csu.edu.cn (T.L.)

**Keywords:** ion channels and transporters, protein language models, multimodal representation learning, protein function prediction, hierarchical classification, Gene Ontology annotation

## Abstract

Ion channels and transporters are diverse membrane proteins that regulate ion flux across biological membranes and play essential roles in cellular signaling, homeostasis, and physiological processes. Accurate classification and functional annotation of these proteins are important for characterizing membrane protein function, interpreting pathogenic mutations, and identifying therapeutic targets. However, classification of ion channels and transporters is complicated by similar fine-grained classes, hierarchical labels, and functional determinants spanning sequence motifs, domains, and structural features. We present IonFusion, which uses sequence-conditioned cross-attention to aggregate domain-derived and structure-aware representations. To model the hierarchical organization of classes of ion channels and transporters, IonFusion employs a coarse-to-fine autoregressive classifier linking five coarse-grained classes to nine fine-grained classes. For GO annotation, IonFusion combines multimodal predictions with DIAMOND homology scores. Through comprehensive benchmarks, IonFusion achieved 0.988 accuracy and 0.860 macro-F1, outperforming eight baselines. Across three GO ontologies and sequence-identity thresholds of ≤10%, ≤30%, and ≤50%, it achieved the highest Fmax in all nine settings (mean 0.732). Ablations supported contributions from each modality and both prediction components. Attention-based and targeted perturbation analyses highlighted a conserved, class-associated motif in potassium channels. IonFusion provides a multimodal computational framework for fine-grained classification of ion channels and transporters and GO annotation within the evaluated label spaces. By integrating complementary multimodal representations with hierarchical prediction and homology-based fusion, IonFusion improves the discrimination of closely related ion channel and transporter classes and enhances GO annotation performance across different sequence-identity thresholds.

## 1. Introduction

Transmembrane ion transport is central to electrochemical homeostasis, cellular excitability and signal transduction [1,2]. These processes are mediated by ion channels and ion transporters, whose activities shape nerve impulse propagation, muscle contraction, hormone secretion and many other physiological processes [3]. Accurate computational classification of these proteins is important for functional annotation, the interpretation of pathogenic mutations, and therapeutic target discovery [4].

However, ion channels and transporters are difficult to classify accurately because their functional identities depend not only on amino acid sequence, but also on conserved domains, transmembrane topology, pore-forming regions, selectivity filters, and local three-dimensional conformations [5,6,7]. Experimental approaches such as patch-clamp recording can directly characterize ion permeability and gating behavior, but their labor and cost requirements limit their scalability to rapidly growing protein sequence databases [8,9,10]. Computational prediction has therefore become an important complement to experimental characterization. Early methods for classification of ion channels and transporters mainly relied on handcrafted sequence features, including amino acid composition, dipeptide (2-mer) composition, and position-specific scoring matrices. These features were combined with models such as support vector machines, multilayer perceptrons, and convolutional neural networks. Representative methods, including VGIchan [11], IonchanPred [12], and DeepIon [13], showed that protein sequences contain discriminative information for classification of ion channels and transporters. However, these methods may not fully capture long-range residue dependencies, conserved functional motifs, and higher-order patterns associated with complex ion channel and transporter families.

Currently, pretrained protein language models (PLMs) have advanced protein representation learning by learning contextual sequence features from large protein sequence corpora [14,15,16]. ESM-2 [17] provides evolutionarily informed sequence embeddings, whereas ESM-3 [18] and SaProt [19] incorporate structural information into protein representations. PLM-based approaches have consequently improved several prediction tasks involving membrane proteins, including ion channels and transporters. For example, DeepPLM_mCNN [20] and TooT-PLM-ionCT [21] combine PLM embeddings with task-specific classifiers for membrane protein classification. However, ion selectivity and subtype specificity are also associated with pore geometry, transmembrane segments, selectivity-filter motifs, conserved residues, and functional domains. Many existing ion channel and transporter classifiers rely primarily on sequence-derived embeddings and do not explicitly model interactions among sequence, domain, and structural information. Integrating multimodal protein information may therefore improve predictive accuracy and interpretability, potentially supporting the directed evolution and rational redesign of ion channels.

Protein structural information provides an important complementary perspective for functional modeling. AlphaFold [22] and related deep learning methods have greatly expanded the availability of predicted protein structures [23]. These structures allow downstream models to incorporate spatial conformations and local structural environments rather than relying solely on primary sequences [24]. Graph-based methods such as DeepFRI [25] and GAT-GO [26] represent protein structures as residue-interaction graphs. They learn structural representations using graph neural networks or attention mechanisms, thereby capturing spatial dependencies that are not explicit in sequence data. For ion channels and transporters, structural information is particularly relevant because selectivity filters, pore regions, and transmembrane conformations are closely associated with ion permeability and subtype specificity.

Another challenge is that labels for ion channels and transporters are not naturally flat. These labels can be organized by ion selectivity, with five coarse-grained classes comprising ion transporters and potassium, calcium, sodium, and chloride channels. Four rarer channel classes (labels 5–8) are mapped to their parent coarse-grained classes. Conventional flat classifiers treat the nine fine-grained classes as independent labels and therefore ignore their relationships to the five coarse-grained classes. This limitation is particularly important for rare fine-grained classes and highly homologous families, for which models must distinguish subtle local differences while preserving broader biological relationships. These characteristics motivate a hierarchy-aware coarse-to-fine strategy in which high-level predictions provide conditional information for autoregressive fine-grained prediction.

Beyond ion-selective classification, multi-label functional annotation is equally important. Gene Ontology (GO) [27] provides a standardized hierarchical framework describing protein functions across the Biological Process Ontology (BPO), Cellular Component Ontology (CCO), and Molecular Function Ontology (MFO). GO-based annotation is particularly relevant for ion channels and transporters, whose functions encompass diverse molecular activities, membrane localization, transport processes, and signaling pathways. Early GO prediction methods transferred annotations based on sequence homology using tools such as BLAST [28] and DIAMOND [29], but their performance often declines for low-similarity or remotely homologous proteins. Recent methods improve prediction by integrating heterogeneous information or modeling label dependencies [30,31,32]. For example, NetGO [33] and MetaGO [34] combine sequence, domain, and interaction features, while DeepGO-based models learn sequence motifs and exploit GO hierarchy constraints for function prediction [35,36,37]. However, these approaches still face challenges for rare and deeply nested GO terms and often incorporate structural constraints only to a limited extent. These limitations can restrict their ability to capture complex structure–function relationships in ion channels and transporters.

To address these limitations, we introduce IonFusion, a multimodal framework for the classification and functional annotation of ion channels and transporters. IonFusion integrates representations of protein sequences, functional domains, and structural conformations through cross-modal attention [38], and employs a hierarchical autoregressive strategy to classify proteins from coarse-grained classes to fine-grained classes. We further extend IonFusion to multi-label Gene Ontology annotation, providing a complementary assessment of its capacity to learn functionally informative representations. Benchmark results show that IonFusion outperforms representative state-of-the-art methods in both the classification and functional annotation of ion channels and transporters. Case studies further indicate that IonFusion can identify biologically relevant sequence motifs.

## 2. Results

### 2.1. Overview of IonFusion

**Figure 1:**
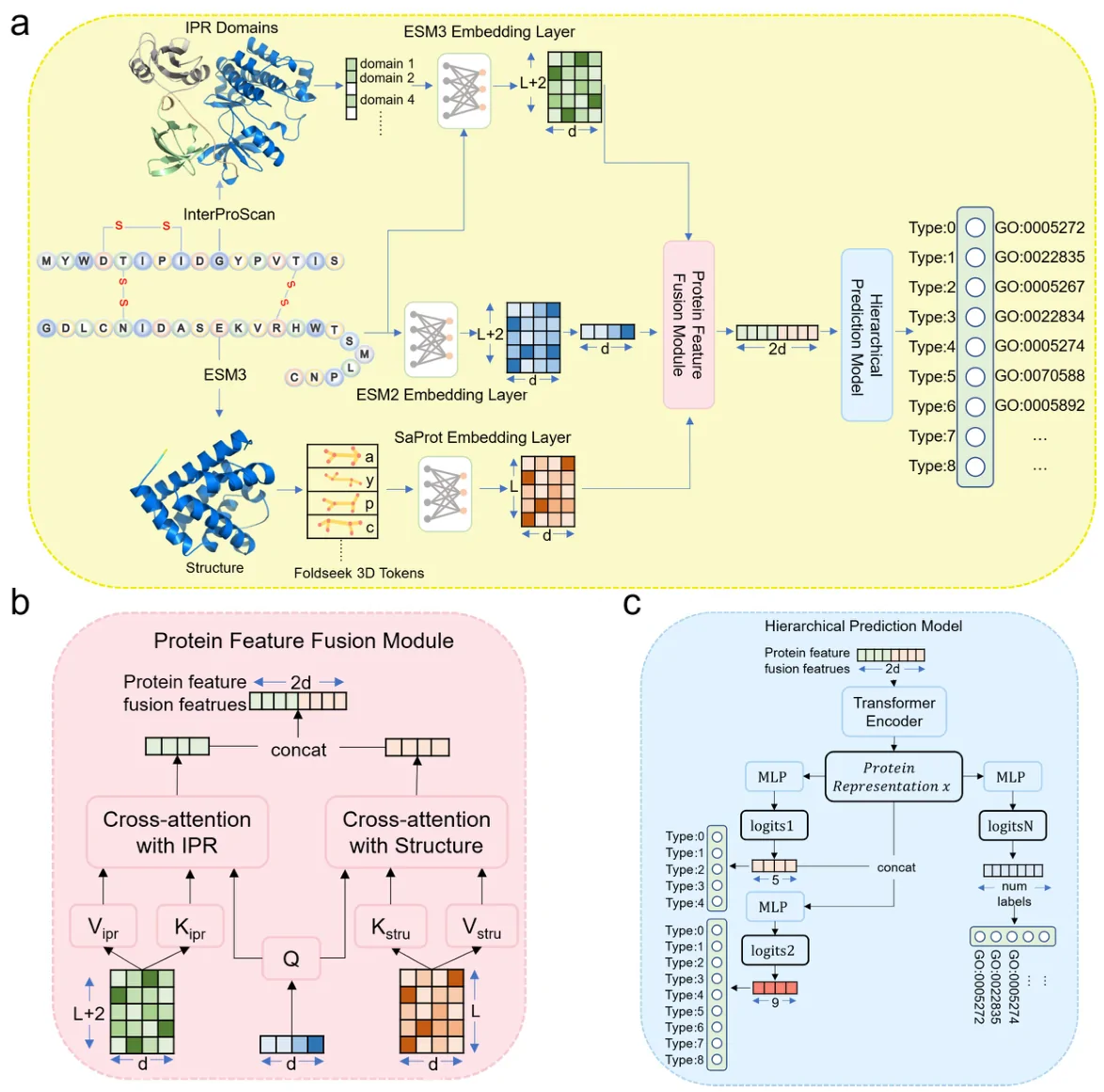
IonFusion model architecture. (**a**) Overview of the framework. IonFusion integrates sequence embeddings from ESM-2, functional features from InterProScan and ESM-3, and structure-aware embeddings generated by SaProt from ESM-3-predicted structures. These heterogeneous features are projected into a unified feature space and then refined through the protein feature fusion module, where cross-attention mechanisms explicitly model interactions among sequence-, function-, and structure-related features. (**b**) Protein feature fusion module. Cross-attention uses the sequence representation to aggregate functional and structural features into a compact multimodal representation. (**c**) Hierarchical prediction module. A Transformer encoder processes the fused representation, and task-specific multilayer perceptron (MLP) heads perform coarse-to-fine ion-selectivity classification and multi-label Gene Ontology (GO) term prediction.

As illustrated in Figure 1, IonFusion processes each protein through five stages. The first step is multimodal feature extraction: ESM-2 produces residue-level sequence embeddings; InterProScan annotations are encoded with ESM-3 to represent functional-domain information; and ESM-3-predicted structures are converted by SaProt into structure-aware embeddings. These features are then aligned through separate projection layers that map the sequence query and the functional and structural key–value streams to a common hidden dimension while retaining their modality-specific content. Next, a global sequence summary serves as the query in two cross-attention operations, with the functional and structural streams serving as keys and values. The two attended outputs are concatenated to form a joint sequence-conditioned representation. For ion-selectivity prediction, a Transformer encoder refines the fused representation. The first classifier predicts five coarse-grained classes, and the fine-grained classifier conditions on the coarse-grained output to produce nine fine-grained class probabilities through the autoregressive hierarchy. For GO annotation, a task-specific head predicts terms in the training-derived GO vocabulary. These probabilities are fused with DIAMOND homology scores using a coefficient selected on the validation set, after which the validation-selected threshold is applied to obtain the predicted GO term set. Section 4 provides the mathematical definitions and implementation details for each stage.

### 2.2. IonFusion Improves Classification of Ion Channels and Transporters

**Figure 2:**
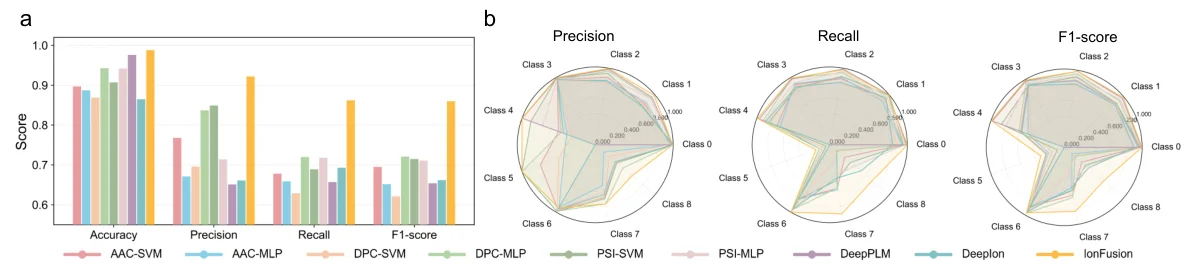
Classification performance comparison between IonFusion and the evaluated methods. Each color represents the same method across both panels, and higher values indicate better performance. (**a**) Overall accuracy and macro-averaged precision, recall, and F1 score. (**b**) Class-wise precision, recall, and F1 score across nine classes: 0, ion transporter; 1, potassium channel; 2, calcium channel; 3, sodium channel; 4, chloride channel; 5, Cys-loop receptor channel; 6, ionotropic glutamate channel; 7, ATP-gated channel; and 8, proton-gated channel. Test-set support for each class is reported in Supplementary Table S2.

We compared IonFusion with eight representative baselines on the large-scale ion-selectivity classification dataset. These baselines included six traditional feature-based classifiers (AAC-SVM, AAC-MLP, DPC-SVM, DPC-MLP, PSI-SVM, and PSI-MLP) and two deep learning methods (DeepPLM and DeepIon). Full results are reported in Supplementary Table S5, and Figure 2a summarizes performance across the four evaluation metrics.

IonFusion achieved the highest macro-averaged precision, recall, and F1 score, with values of 0.922, 0.862, and 0.860, respectively. Its overall accuracy was 0.988. Supplementary Table S5 additionally reports support-weighted precision, recall, and F1 score, which were 0.989, 0.988, and 0.989, respectively. The difference between macro-F1 and weighted F1 reflects the influence of the well-represented classes in this imbalanced dataset and highlights the complementary information provided by the two averaging strategies. Overall, IonFusion outperformed the evaluated baseline methods across the nine-class classification task.

IonFusion exceeded the accuracy of the second-best deep-learning baseline, DeepPLM, by 1.2%. Consistent with this improvement, IonFusion also achieved the highest support-weighted precision, recall, and F1 score among the evaluated methods, with absolute gains of 2.3%, 1.2%, and 1.8% over DeepPLM, respectively. The improvements in both support-weighted and macro-averaged metrics suggest that combining sequence, functional, and structural representations was beneficial under this evaluation setting.

The advantage of IonFusion was also evident in macro-averaged precision, recall, and F1 score. Its macro-F1 score exceeded that of the best traditional baseline, DPC-MLP, by 13.9%. The corresponding differences relative to DeepIon and DeepPLM were 19.8 and 20.6%, respectively. IonFusion achieved a macro-recall of 0.862, compared with 0.629–0.720 for the evaluated baselines, an absolute gain of 14.2–23.3%. Overall, IonFusion achieved the balance between macro-precision and macro-recall among the evaluated methods on this imbalanced nine-class task.

Ion channel families often exhibit strong evolutionary conservation and substantial sequence homology. For instance, sodium and calcium ion channel proteins share similar sequences, and Cys-loop receptor ion channel proteins are related to chloride channel proteins. Such similarities complicate fine-grained ion-selectivity classification for conventional methods. To assess fine-grained class discrimination, we compared the class-wise precision, recall, and F1 scores of IonFusion with those of all baseline methods. The complete results are reported in Supplementary Table S6.

Across the nine classes, IonFusion achieved the highest F1 score among the evaluated methods in eight of the nine classes (Figure 2b). The only exception was the ionotropic glutamate receptor channel class (label 6), for which IonFusion achieved an F1 score of 0.966, slightly below the 0.973 obtained by DPC-MLP. Differences among methods were also apparent in several underrepresented classes, where some baseline models showed substantial performance degradation and, in some cases, F1 scores of 0.000. For Cys-loop receptor channels (label 5), IonFusion achieved the highest F1 score among the compared methods at 0.320, although its recall was only 0.190 and the test set contained just 21 proteins. Similarly, for proton-gated channels (label 8), IonFusion achieved the highest F1 score of 0.664 on 116 test proteins. Overall, the class-wise results show that IonFusion achieved higher F1 scores than the evaluated baselines in most classes, including several underrepresented subclasses. However, the modest absolute performance and limited sample support for the rarest subclasses, particularly Cys-loop receptor channels, indicate that prediction of these fine-grained classes remains challenging.

### 2.3. Ablation of Feature Modalities and Prediction Components

To quantify the contributions of the sequence, functional, and structural feature modalities and of the hierarchical and autoregressive prediction components, we conducted ablation experiments on the same dataset. We evaluated the complete IonFusion model and six variants: a model without sequence embeddings (IPR+Str), a model without functional embeddings (Seq+Str), a model without structural embeddings (Seq+IPR), a sequence-only model (Seq), a model without hierarchical classification (NoLayer), and a model without autoregressive prediction (NoAR). The macro-averaged results are reported in Supplementary Table S7.

**Figure 3:**
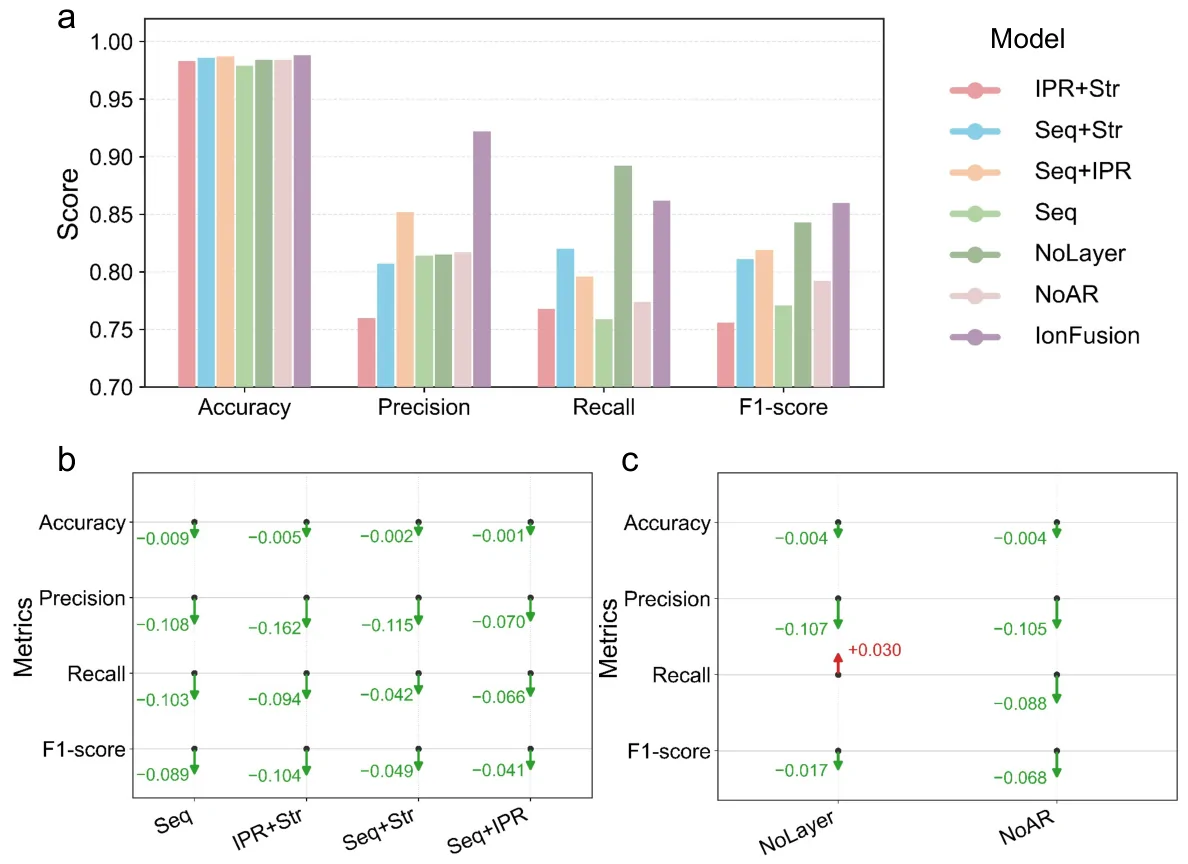
Ablation study of IonFusion components and architecture. Each reference point corresponds to the performance of the full IonFusion model under the same evaluation setting. Green markers indicate performance degradation after removing specific components, whereas red markers indicate performance improvement. (**a**) Overall comparison of model variants across accuracy, precision, recall, and F1-score. (**b**) Effects of progressive feature ablation (sequence, IPR, and structure), where metric changes are visualized as dumbbell plots relative to the full model. (**c**) Effects of architectural ablation, including removal of the layered structure (NoLayer) and autoregressive module (NoAR), with corresponding metric deviations from the full model shown in dumbbell plots.

Overall, the complete IonFusion model achieved the highest accuracy, precision, and F1 score among all evaluated variants, despite a slightly lower recall than NoLayer. Figure 3a indicates that integrating sequence, functional, and structural information with hierarchical and autoregressive prediction provided a better overall balance across the evaluated classification metrics.

#### 2.3.1. Contribution of Feature Modalities

Removing any single modality reduced at least one performance metric, indicating that all three modalities contributed to the classification task. This trend is most evident in Figure 3b. Excluding sequence embeddings produced the largest overall decrease: macro-averaged precision, recall, and F1 score declined by 16.2%, 9.4%, and 10.4%, respectively. Among the three modalities, sequence representations therefore provided the strongest individual contribution to ion-selectivity classification.

When functional embeddings were removed, accuracy and recall remained relatively high, whereas macro-precision decreased by 11.5%. Omitting structural embeddings reduced macro-recall by 6.6% while accuracy and macro-precision remained comparatively stable. These changes support complementary contributions from the functional and structural representations under the evaluated setting.

When sequence embeddings alone were used, accuracy, macro-precision, macro-recall, and macro-F1 decreased by 0.9%, 10.8%, 10.3%, and 8.9%, respectively, compared with the complete model. Sequence features alone therefore retained considerable predictive information, whereas the functional and structural representations provided complementary gains for distinguishing fine-grained classes of ion channels and transporters.

#### 2.3.2. Contribution of Hierarchical and Autoregressive Prediction

We next evaluated the contributions of the hierarchical classification and autoregressive prediction components through ablation experiments (Figure 3c). Compared with the complete IonFusion model, removing the hierarchical classification component (NoLayer) increased macro-recall from 0.862 to 0.892, but reduced macro-precision from 0.922 to 0.815, together with decreases in accuracy and macro-F1 (Supplementary Table S7). The confusion matrices in Supplementary Figure S7 further clarify the source of this precision–recall trade-off. For the Cys-loop receptor class, NoLayer correctly identified 11 of the 21 true samples, corresponding to a class-specific recall of 0.524, whereas the complete model identified 4 of 21 samples (recall = 0.190). However, this gain in sensitivity was accompanied by a substantial increase in false-positive predictions: NoLayer incorrectly assigned 32 ionotropic glutamate receptor channels to the Cys-loop receptor class, whereas no such misclassification was observed for the complete model. Consequently, the precision of Cys-loop receptor predictions decreased from 1.000 with IonFusion to 0.250 with NoLayer. A similar pattern was observed for other relatively small classes. For example, the precision of ATP-gated channel predictions decreased from 0.771 to 0.667, and that of proton-gated channel predictions decreased from 0.619 to 0.486 after removing the hierarchical component. These results indicate that the higher macro-recall of NoLayer was achieved at the cost of substantially more false-positive assignments, ultimately leading to lower macro-precision and macro-F1.

More importantly, the distribution of these errors was consistent with the hierarchical decision structure illustrated in Supplementary Figure S1. Several prominent errors produced by the flat NoLayer model occurred between classes belonging to different predefined decision domains. For example, NoLayer misclassified 32 ionotropic glutamate receptor channels from Decision Domain 1 as Cys-loop receptor channels in Decision Domain 4, whereas this cross-domain error was completely eliminated by the hierarchical IonFusion model. Similar reductions were observed for potassium-to-calcium channel misclassification, which decreased from 20 cases with NoLayer to 2 cases with IonFusion, and for ion transporters misclassified as potassium or calcium channels, which decreased from 17 and 11 cases, respectively, to 0. When the nine classes were further aggregated according to the five predefined decision domains, the total number of cross-domain errors decreased from 191 for NoLayer to 145 for the complete model, corresponding to a 24.1% reduction. Thus, although hierarchical classification reduced sensitivity for some rare classes, it substantially constrained erroneous assignments across biologically defined decision domains and suppressed cross-domain false positives. Together with the increase in macro-precision and macro-F1, these results provide empirical support for the utility of the predefined hierarchical organization in guiding the model toward more discriminative class boundaries.

Removing the autoregressive prediction component (NoAR) consistently degraded performance across all four evaluation metrics. Relative to the complete IonFusion model, NoAR decreased accuracy, macro-precision, macro-recall, and macro-F1 by 0.4%, 10.5%, 8.8%, and 6.8%, respectively (Figure 3c). The particularly pronounced reductions in macro-precision, macro-recall, and macro-F1 indicate that incorporating coarse-grained predictions into the subsequent fine-grained classification stage contributes to more reliable discrimination among the nine protein classes.

Together with the feature-modality ablations, these results show that all three modalities and both prediction components contributed to the performance of IonFusion. Removing sequence embeddings produced the largest overall reduction, whereas the functional and structural representations provided complementary improvements. Hierarchical classification improved precision and overall performance despite a modest reduction in recall, while autoregressive prediction improved all four metrics relative to NoAR. Overall, the complete IonFusion model provided the strongest balance among accuracy, macro-precision, macro-recall, and macro-F1 across the evaluated ablation settings.

### 2.4. Case Studies

Beyond ion-selectivity classification, we examined sequence regions associated with IonFusion’s predictions at the model level. Cross-attention analysis highlighted regions overlapping known motifs, providing a qualitative view of the patterns to which the model assigned higher attention.

**Figure 4:**
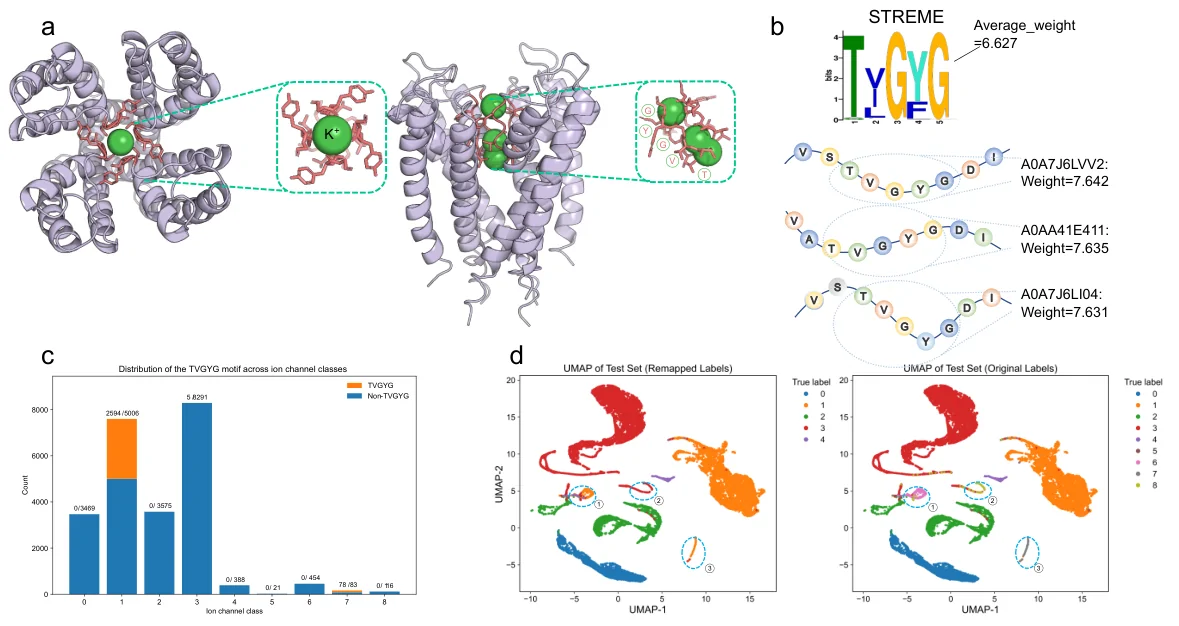
Structural and sequence-level analysis of the TVGYG selectivity filter in potassium channels. (**a**) Structure of the KcsA potassium channel (PDB ID 1BL8) [6,44], with the selectivity filter highlighted. Bound K^+^ ions are shown as green spheres, and the enlarged view at right shows the conserved TVGYG residues forming the filter. (**b**) STREME motif logo for TVGYG and three selected correctly predicted proteins with their reported motif-associated statistics. (**c**) Distribution of the TVGYG motif across the nine test-set ion-selectivity classes. The stacked bars show the numbers of motif-containing sequences (TVGYG, orange) and motif-free sequences (Non-TVGYG, blue) in each class. The labels above the bars report the corresponding TVGYG/Non-TVGYG sequence counts. (**d**) UMAP visualization of learned protein representations in the test set, comparing the five coarse-grained classes (left) with the nine fine-grained classes (right). The blue dashed circles numbered 1–3 highlight corresponding local regions before and after coarse-grained label remapping.

For instance, potassium channels frequently contain a highly conserved TVGYG selectivity-filter motif [6,39]. Electrophysiological measurements of Shaker channels established preferential K^+^ conduction, and mutations at selected positions in their conserved signature sequence altered ion selectivity [39,40]. Crystal structures of KcsA showed that backbone carbonyl oxygens in the selectivity filter form K^+^-coordination sites within the narrow pore [6,41]. Measurements of ion conduction together with crystallographic analysis of ion occupancy further linked the filter to rapid K^+^ permeation [42]. Together, these functional and crystallographic experiments support the TVGYG-containing selectivity filter as a structural determinant of K^+^ selectivity. Figure 4a shows the spatial location of this selectivity filter in the KcsA structure.

We next conducted a descriptive motif analysis of correctly classified potassium-channel proteins. Motif discovery using STREME [43] identified TVGYG as the highest-ranked conserved pattern, as shown in Figure 4b. Of the 7515 correctly classified potassium-channel samples, 2553 contained the exact TVGYG motif. Across these motif-containing sequences, TVGYG had a mean attention-derived score of 6.627, indicating that this conserved region was frequently assigned high importance by the model. In 1710 proteins, exact TVGYG was itself the highest-attention five-residue window, with a mean attention-derived score of 6.665. We further examined pairwise sequence identity among the three selected proteins with the highest motif-associated scores in Figure 4b. Calcium-activated potassium channel subunit alpha-1 (UniProt ID: A0A7J6LVV2) and a potassium channel family protein (UniProt ID: A0AA41E411) shared only 10.32% sequence identity. Despite their low global sequence identity, the conserved TVGYG motif received high motif-associated scores in these examples. Together, these cases collectively demonstrate that IonFusion can allocate high attention weights to discriminative local subsequences to a certain extent, capturing invariant feature representations even in the presence of extreme global sequence divergence.

Because selectivity-filter motifs may tolerate limited sequence variation, we defined TVGYG-like sequences as the exact TVGYG motif or sequences with one substitution at positions 1–4 while retaining the terminal glycine. Mutations at this terminal glycine have been reported to disrupt stable tetramer assembly and abolish detectable channel activity [45]. Under this criterion, 2975 of the 7515 correctly classified potassium-channel proteins (39.59%) contained a TVGYG-like sequence as their highest-attention five-residue window. The six most frequent highest-attention windows are summarized in Supplementary Table S10. These results indicate that, in a substantial subset of correctly classified potassium channels, the model assigns its strongest local attention to the canonical TVGYG motif or to closely related variants.

To further evaluate these six high-attention motifs, Supplementary Figure S8 summarizes their occurrence and attention-derived scores across the entire potassium-channel test set. For each motif, the upper panel reports the number of proteins containing the corresponding exact five-residue sequence. When multiple occurrences of the same motif were present within a protein, only the occurrence with the highest attention-derived score was retained, and the lower panel reports the mean score across all motif-containing proteins. Among the six motifs, TVGYG ranked highest in both measures: it was detected in 2553 proteins and had the highest mean attention-derived score of 6.583. Thus, TVGYG was not only the most frequently observed motif among the selected high-attention patterns, but also showed the strongest average attention signal, further supporting its prominence in the model’s potassium-channel predictions.

We then examined how broadly TVGYG-like sequences occurred across the complete test set of all nine classes (Figure 4c). Exact TVGYG occurred in 2594 of 7600 potassium-channel proteins (34.13%), while allowing one substitution at positions 1–4 with the terminal G fixed increased the coverage to 5165 of 7600 proteins (67.96%) (Supplementary Table S11). Thus, approximately two-thirds of the potassium-channel proteins contained either the canonical TVGYG motif or a closely related five-residue variant somewhere in their full-length sequence. Notably, ATP-gated channels also exhibited a high prevalence of TVGYG-like patterns, with 48.45% containing exact TVGYG and 77.02% containing TVGYG or a one-substitution variant. In contrast, among the remaining seven classes, exact TVGYG was nearly absent: the highest prevalence was only 0.06%, with a mean prevalence of approximately 0.01% across these classes. Even when one substitution was allowed, the highest prevalence among these seven classes was 3.87%, with a mean of only 0.94%.

The high prevalence of TVGYG-like patterns in both potassium and ATP-gated channels indicates that these two classes share substantial local sequence similarity. This observation provides support for the hierarchical organization defined in Supplementary Figure S1, in which ATP-gated channels and potassium channels are placed within closely related branches. The shared enrichment of TVGYG-like patterns therefore suggests that the predefined hierarchy captures meaningful sequence-level relationships between these channel classes.

To test whether the attention-highlighted TVGYG signal contributed to the model output, we performed perturbation analysis on 2553 potassium-channel proteins that were originally correctly classified and contained an exact TVGYG motif (Supplementary Figure S9). Following perturbation, the potassium-channel model score decreased in 2070 of 2553 proteins (81.08%), increased in 360 (14.10%), and remained unchanged in 123 (4.82%). At the prediction level, 793 proteins (31.06%) were no longer classified as potassium channels and were instead reassigned to one of the other eight classes. This effect was more pronounced among the 1710 proteins in which TVGYG was the highest-attention five-residue window: 620 proteins (36.26%) were reassigned after perturbation, whereas 1090 (63.74%) remained classified as potassium channels. In comparison, among the other 843 TVGYG-containing proteins, 173 (20.52%) were reassigned and 670 (79.48%) remained potassium-channel predictions. Thus, perturbation of the TVGYG region not only reduced the potassium-channel score in the majority of proteins but also altered the final predicted class in nearly one-third of the analyzed proteins, with a substantially higher reassignment rate when TVGYG corresponded to the highest-attention window. These results provide model-level evidence that the TVGYG region contributes to the model’s potassium-channel classification decisions.

Furthermore, we used UMAP [46] to project the multimodal fused representations of test-set proteins into two dimensions, detailed in (Figure 4d). The left panel colors the samples according to five coarse-grained labels, whereas the right panel uses the original nine fine-grained labels. Several coarse-grained classes formed visually coherent regions in the projected space.

A closer inspection of the right panel shows that several rare ligand-gated fine-grained classes with small sample sizes, such as label 5 Cys-loop receptors, label 6 ionotropic glutamate receptors, label 7 ATP-gated channels, and label 8 proton-gated channels, do not form completely isolated clusters in the two-dimensional latent space. Instead, they show obvious proximity or even manifold overlap with certain majority classes, such as label 1 potassium channels, label 3 sodium channels, and label 4 chloride channels, in local boundary regions. These local regions are marked by circles 1–3 in the figure. This observation suggests that, at the fine-grained level, these ion channel classes exhibit substantial similarity in sequence patterns, domain composition, or structural features. Therefore, directly performing flat nine-class classification may increase the risk of class confusion.

However, after coarse-grained label remapping, the fine-grained overlaps observed in the right panel are reassigned in the left panel to coarse-grained classes defined by the hierarchical classification strategy proposed in this study. Thus, the local overlaps highlighted by Circles 1–3 do not represent arbitrary misclassification; rather, they reflect the intrinsic functional similarity among these fine-grained classes at the coarse-grained level. This pattern is consistent with the underlying logic of the proposed framework: the model first learns coarse-grained classes characterized by relatively stable decision boundaries and subsequently discriminates among fine-grained ion-selectivity classes within the same or closely related higher-level categories.

Overall, UMAP provided a descriptive view of class proximity in the learned representation space. The correspondence between the coarse-grained and fine-grained labels in Circles 1, 2, and 3 is compatible with the predefined hierarchy. Compared with direct flat fine-grained classification, the proposed coarse-to-fine prediction mechanism is better suited to capturing the predefined relationships and local similarities among classes of ion channels and transporters.

### 2.5. IonFusion Extends to GO Annotation

To further evaluate IonFusion for multi-label GO term prediction, we compared it with five representative baselines on datasets constructed at sequence-identity thresholds of 10%, 30%, and 50%. The comparison methods included the PLMs ESM-2 and ESM-3, the domain annotation method InterPro, and the GO prediction methods DeepGOZero and DeepGOSE. Performance was evaluated separately within the BPO, CCO, and MFO namespaces. Full results across all sequence-identity thresholds and GO namespaces are reported in Supplementary Table S8.

#### 2.5.1. Overall Performance Analysis

**Figure 5:**
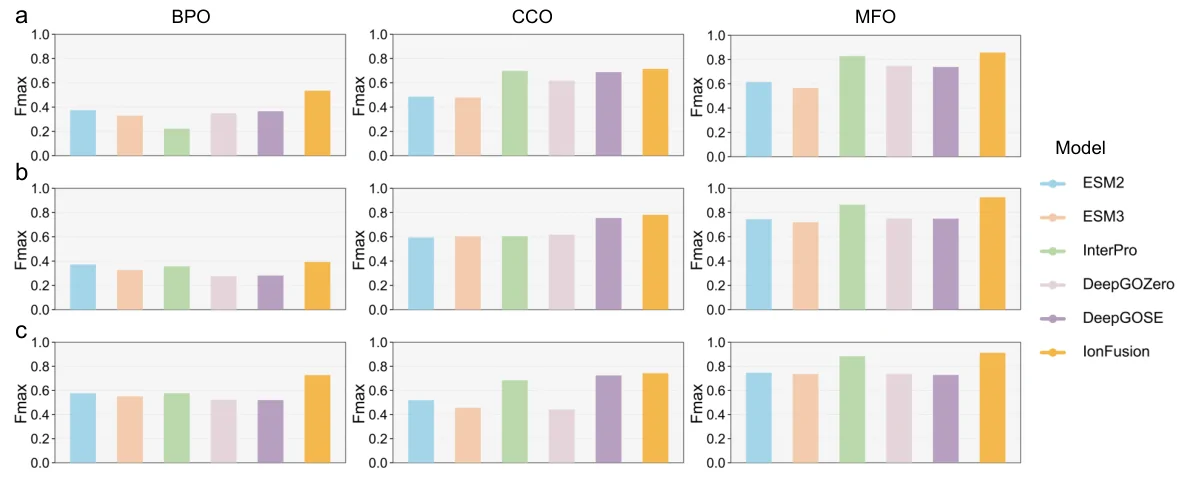
Fmax comparison for Gene Ontology (GO) function prediction. Columns correspond to BPO, CCO, and MFO. (**a**–**c**) show separately constructed datasets with sequence-identity thresholds of ≤10%, ≤30%, and ≤50%, respectively. Higher values indicate better performance within each panel.

Figure 5 shows that IonFusion achieved the highest Fmax across all nine combinations of sequence-identity threshold and GO namespace. Its mean Fmax was 0.732, with absolute differences of 0.174, 0.203, and 0.097 relative to ESM-2, ESM-3, and InterPro, respectively, and differences of 0.170 and 0.115 relative to DeepGOZero and DeepGOSE. These results support the utility of integrating sequence, domain, structural, and DIAMOND-derived homology information within the evaluated datasets. Notably, although IonFusion consistently achieved the highest Fmax within each evaluation setting, the absolute Fmax values did not vary monotonically with increasing sequence-identity thresholds. This pattern was not specific to IonFusion: each evaluated method exhibited at least one non-monotonic trend across BPO, CCO, or MFO (Supplementary Table S8). In BPO, for example, five of the six methods—IonFusion, ESM-2, ESM-3, DeepGOZero, and DeepGOSE—showed lower Fmax at the 30% setting than at the 10% setting, followed by an increase at the 50% setting, with InterPro being the only exception. IonFusion’s corresponding BPO Fmax values were 0.535, 0.393, and 0.727 at thresholds of ≤10%, ≤30%, and ≤50%, respectively. This non-monotonic behavior can be understood from the construction of the evaluation datasets: the three threshold-specific datasets were generated independently and therefore contain different protein and GO-term compositions, rather than forming nested subsets or repeated evaluations of an identical test cohort. Consequently, increasing the sequence-identity threshold does not necessarily lead to monotonic improvement in Fmax, and the more appropriate comparison is between methods evaluated under the same threshold and GO namespace.

To complement this comparison and further examine model performance under different levels of sequence similarity within a common evaluation framework, we conducted an additional nested-subset analysis using the original 50%-identity test set (Supplementary Table S12). For each test protein, MMseqs2 was used to calculate its maximum sequence identity to the training set, and proteins with maximum identities below 10% and 30% were selected to form two nested low-identity subsets. The complete original test set was retained as the corresponding <50% group. In contrast to the independently constructed datasets used in the main comparison, these three subsets were derived from the same original 50% test set and therefore provide a complementary view of model performance across increasing sequence-identity ranges.

Across these nested subsets, IonFusion showed a consistent increase in performance as the permitted maximum sequence identity to the training set increased from <10% to <30% and <50%. For BPO, Fmax increased from 0.445 to 0.506 and 0.727 across the <10%, <30%, and complete original test sets, respectively. Similar trends were observed for CCO, where Fmax increased from 0.517 to 0.553 and 0.743, and for MFO, where it increased from 0.716 to 0.793 and 0.912. AUPR, ICAUPR, and DPAUPR exhibited comparable upward trends. The nested-subset analysis complements the comparisons based on the independently constructed 10%, 30%, and 50% datasets.

IonFusion also performed strongly for AUPR (Supplementary Figure S2), ICAUPR (Supplementary Figure S3), and DPAUPR (Supplementary Figure S4). Its average AUPR was 0.673, exceeding those of ESM-2, ESM-3, InterPro, DeepGOZero, and DeepGOSE by 14.1%, 15.7%, 18.0%, 17.6%, and 6.9%, respectively. These gains indicate that IonFusion maintained a favorable precision–recall balance despite the long-tailed distribution of GO labels.

Similar trends were observed for ICAUPR and DPAUPR, with IonFusion achieving the highest or near-highest scores in most settings. Its consistent performance on these metrics indicates effective prioritization of information-rich and hierarchically deep GO terms. Within the CCO namespace, however, DeepGOSE achieved slightly higher AUPR-based scores in some settings. This pattern may indicate that explicit ontology propagation or hierarchy-aware semantic reasoning is particularly beneficial for cellular component annotation.

Overall, across different dataset splits, the aggregated results for AUPR, ICAUPR, and DPAUPR showed that IonFusion achieved the highest average performance compared to other baseline methods, indicating its performance in different data partition settings.

#### 2.5.2. Analysis of Deep, Highly-Specific Function Prediction Capability

To assess IonFusion’s ability to predict deep and functionally specific GO terms, we calculated label-wise AUPR for terms with a depth greater than 6. The analysis was performed at three sequence-identity thresholds (≤10%, ≤30%, and ≤50%) and is summarized in Supplementary Figure S5. Within each GO namespace, greater term depth indicates a larger distance from the corresponding root and generally corresponds to greater functional specificity. Accordingly, restricting the evaluation to GO terms with depth >6 provides a focused assessment of performance on fine-grained functional annotations.

IonFusion showed favorable label-wise AUPR performance for GO terms with depth >6 at all three sequence-identity thresholds, although its margin over the baselines varied among settings. At the ≤50% threshold (Supplementary Figure S5c), IonFusion consistently outperformed the compared methods across BPO, CCO, and MFO. Under the stricter thresholds (≤10% and ≤30%, Supplementary Figure S5a,b), its AUPR distributions were generally higher than or comparable with those of the baselines.

The separation between IonFusion and the baselines became more pronounced at the ≤50% threshold, where its AUPR distributions shifted upward across all three GO namespaces. IonFusion also remained among the best-performing methods in the two lower-identity datasets. Overall, these results indicate that IonFusion maintained strong comparative performance across datasets constructed using different sequence-identity thresholds.

#### 2.5.3. Generalization Performance Under Varying Homology Constraints

Across the sequence-identity thresholds shown in Supplementary Figure S6, IonFusion maintained competitive Fmax and AUPR scores. At the ≤10% threshold, the method remained the best-performing comparator within each GO namespace. This result supports performance on the corresponding low-identity evaluation sets.

As the threshold increased from ≤10% to ≤50%, several baselines improved, whereas IonFusion retained the highest overall mean performance across all four evaluation metrics. This pattern is consistent with complementary contributions from multimodal representations and homology-search information.

Supplementary Table S9 quantifies the fixed-vocabulary boundary of GO prediction. Out-of-vocabulary (OOV) terms comprised 0.32–7.41% of propagated test annotation occurrences across the nine threshold–ontology settings, although 2.95–33.53% of proteins had at least one OOV annotation. The missing-label fraction is therefore modest at the annotation level but affects more proteins in some settings, particularly BPO. Because the prediction vocabularies were defined by the corresponding training splits, this is a shared limitation of the evaluated fixed-vocabulary task rather than an IonFusion-specific error.

Overall, these results show that IonFusion is applicable not only to fine-grained classification of ion channels and transporters but also to multi-label GO term prediction within the training-defined GO label space. Across the evaluated settings, the combined use of sequence, domain, structural, and DIAMOND-derived homology information was associated with improved GO term prediction.

## 3. Discussion

We developed IonFusion, a multimodal hierarchical framework that integrates sequence, functional, and structural representations to classify ion channels and transporters and predict GO terms. Cross-attention learns interactions among these modality-specific representations and constructs a joint representation for downstream prediction. An autoregressive hierarchy then links predictions for five coarse-grained classes to nine fine-grained classes. Together, these components address heterogeneous feature integration and structured coarse-to-fine prediction.

On the classification dataset, IonFusion achieved higher macro-averaged precision, recall, and F1 score than the evaluated baselines. Removing any one modality weakened at least one metric and generally reduced overall performance, supporting complementary contributions from the three input branches. However, performance was uneven across classes. The low F1 score for Cys-loop receptor channels and the moderate result for proton-gated channels show that the model remains sensitive to extreme class imbalance. The confusion-matrix analysis further showed that the hierarchy mainly suppressed cross-domain false positives, improving macro-precision while sacrificing some recall (Supplementary Figure S7). TVGYG was a recurrent high-attention motif, and perturbing it changed the predicted class for 31.06% of the analyzed motif-containing proteins (Supplementary Figures S8 and S9).

Extending IonFusion to multi-label GO prediction tested whether the fused representations were useful beyond ion-selectivity classification. The combined multimodal and homology-search pipeline achieved the highest overall mean performance across the four GO metrics, although individual baselines remained competitive in some settings (Supplementary Table S8). The shared OOV label space in Supplementary Table S9 also limits the attainable recall.

Analysis of GO terms with depth >6 provided a more focused assessment of relatively specific functional annotations. Across the evaluated thresholds, IonFusion’s label-wise AUPR distributions were generally higher than or comparable with those of the baselines, with clearer separation at ≤50%. This pattern suggests that the complete model retained predictive performance on hierarchically deep terms under the evaluated conditions.

Because the GO label space was defined by annotations in the training split, IonFusion cannot output previously unseen GO terms; the same boundary applies to the compared fixed-vocabulary methods (Supplementary Table S9). This reflects incomplete annotation coverage in protein function prediction rather than an IonFusion-specific error. Retaining OOV ground-truth terms in the recall denominator avoids an optimistic evaluation but limits attainable recall. Open-vocabulary or ontology-aware prediction remains a necessary test of transfer to new functional labels.

Overall, these results support IonFusion as a computational framework for fine-grained classification of ion channels and transporters and multi-label GO term prediction. The framework may also provide a foundation for broader protein function annotation, although its transferability to additional protein families and independently curated datasets warrants further investigation. Future work could extend the current multimodal framework by incorporating experimentally resolved protein structures, protein–protein interaction networks, and explicit evolutionary conservation profiles, which may provide complementary information for functional characterization. In addition, evaluation on independently curated datasets, additional protein families, and expanded or temporally updated GO vocabularies will help further assess the robustness and transferability of IonFusion. More systematic analyses of the contributions and interactions of different information sources may also provide further insight into how multimodal representations support protein function prediction.

## 4. Materials and Methods

### 4.1. Data Collection and Processing

The dataset used in this study was primarily obtained from UniProt [47]. Supplementary Table S1 summarizes the 250,882 records before quality control: 50,000 ion transporter records, 200,788 records assigned to eight ion channel classes, and 94 records with a Null label. Quality control removed all 94 Null-labelled records and 1605 records with abnormal sequence lengths, incomplete annotations, or clear annotation errors. These exclusions left 249,183 proteins in the curated dataset.

Based on biological function and ion selectivity, the proteins were organized using the hierarchical labelling scheme shown in Supplementary Figure S1. At the coarse-grained level, proteins were assigned to classes 0–4 according to prior biological knowledge. The hierarchy was refined by introducing labels 5–8, yielding nine classification labels in total.

The classification and GO annotation tasks used separate CD-HIT workflows. For the nine-class classification task, the 249,183 quality-controlled sequences were clustered with CD-HIT [48] at a 40% sequence-identity threshold before partitioning. All members of each cluster were assigned to the same split. No CD-HIT cluster was shared between the training and test sets. We then retained proteins for which sequence, structure, and text embeddings were all available. This step excluded 10,612 training records and 2831 test records, leaving 211,660 training and 24,080 test proteins for model training and evaluation, respectively. Supplementary Table S2 reports the class distributions of these final, embedding-complete sets.

For the GO task, we independently intersected the 235,740 ion channel and transporter sequences with 573,661 UniProt proteins carrying experimentally supported annotations [47]. This yielded 3131 proteins with GO annotations. Before constructing the training-derived prediction vocabularies, the combined annotation set contained 2456 BPO terms, 425 CCO terms, and 444 MFO terms. Starting from this GO-annotated subset, we ran CD-HIT separately at sequence-identity thresholds of 10%, 30%, and 50% to construct three independent datasets. At each threshold, proteins were clustered before partitioning, and all members of a cluster were assigned to one training, validation, or test set, targeting an approximately 8:1:1 split. These threshold-specific procedures were separate from the 40% classification workflow and were not successive filters. Within each threshold, no CD-HIT cluster or protein identifier was shared across partitions. The three splits contained the same numbers of proteins but differed in protein and GO-term composition, as summarized in Supplementary Table S3.

### 4.2. Multimodal Feature Generation

To capture complementary biological information about ion channels and transporters, IonFusion first uses InterProScan to extract InterPro (IPR) annotations from input sequences [49,50], ESM-2 to generate sequence embeddings, ESM-3 to encode functional information and predict three-dimensional protein structures, and SaProt to generate structure-aware embeddings from the predicted structures.

#### 4.2.1. Sequence-Level Feature Extraction

IonFusion uses ESM-2 (esm2_t33_650M_UR50D) as the backbone encoder to generate residue-level contextual representations, denoted as $E_{\mathrm{seq}}\in R^{1\times (L+2)\times 1280}$, where *L* is the protein sequence length. These features retain residue order and characterize contextual dependencies between residues and potential long-range interaction patterns.

#### 4.2.2. Function-Level Feature Extraction

To capture functional variation among ion channels and transporters, IonFusion uses InterProScan to derive InterPro (IPR) annotations which are subsequently encoded by ESM-3. The resulting functional embeddings are denoted as $E_{\mathrm{ipr}}\in R^{1\times (L+2)\times 1536}$. These embeddings encode attributes such as domain composition and conserved-site annotations, complement the primary-sequence representation, and are supplied to the cross-modal attention module.

#### 4.2.3. Structure-Aware Feature Extraction

Based on the structures predicted by ESM-3, IonFusion uses SaProt to generate structure-aware embeddings, denoted as $E_{\mathrm{stru}}\in R^{1\times L\times 1280}$. These embeddings represent local spatial conformations and associated structural patterns and are subsequently provided to the cross-modal attention module. Because ion selectivity and function are closely associated with local structural environments, these structure-aware features may expose discriminative cues that are obscured in primary sequences alone.

### 4.3. Multimodal Feature Alignment and Hierarchical Prediction

Because sequence, functional, and structural representations differ in dimensionality and occupy distinct semantic spaces, direct concatenation may yield poorly aligned features. IonFusion therefore uses a cross-attention fusion module to align and adaptively weight the three modalities. The resulting fused representation is processed by a transformer encoder and a hierarchical autoregressive prediction head for coarse-to-fine ion-selectivity classification.

#### 4.3.1. Cross-Modal Feature Alignment and Fusion

Given $E_{\mathrm{seq}}$, $E_{\mathrm{ipr}}$, and $E_{\mathrm{stru}}$, the initial position token of the sequence embedding is used as the global sequence summary, denoted as $E_{\mathrm{token}}\in R^{1\times 1280}$.

To align the three modalities within a shared semantic space and capture their underlying correlations, the feature tensors are first projected into the same hidden dimension *d* through learnable linear projections, where d=1280. Specifically, the sequence global summary $E_{token}$ is mapped into a query vector (Query,q):

(1) $$q=\mathrm{LayerNorm}\left(E_{\mathrm{token}}W_{q}\right)\in R^{1\times 1280}$$

Similarly, the functional embeddings $E_{ipr}$ and structural embeddings $E_{stru}$ are mapped into key vectors (Key,k) and value vectors (Value,v):

(2) $$k_{i}=\mathrm{LayerNorm}\left(E_{i}W_{k,i}\right)\in R^{d_{i}\times 1280}$$

(3) $$v_{i}=\mathrm{LayerNorm}\left(E_{i}W_{v,i}\right)\in R^{d_{i}\times 1280}$$

where i∈{ipr,stru}, $d_{ipr}=L+2$, and $d_{stru}=L$. $W_{q}$, $W_{k,i}$, and $W_{v,i}$ denote the learnable weight matrices during model training. LayerNorm represents the layer-normalization operation, which is designed to accelerate convergence and stabilize the training procedure.

Subsequently, with the sequence global summary *q* as a guide, cross-attention weights $\alpha_{i}$ are computed between the sequence summary and each position in the functional and structural embeddings. These weights parameterize the sequence-conditioned aggregation of the functional and structural representations:

(4) $$\alpha_{i}=\mathrm{softmax}\left(\frac{q\cdot k_{i}^{T}}{\sqrt{d_{k}}}\right)\in R^{1\times d_{i}}$$

The resulting attention weights are then used to compute dynamic weighted sums over corresponding value vectors, yielding aligned fused representations $y_{i}$ for each modality:

(5) $$y_{i}=\alpha_{i}\cdot v_{i}\in R^{1\times 1280}$$

Finally, the aggregated functional features $y_{\mathrm{ipr}}$ and structural features $y_{\mathrm{stru}}$ are concatenated along the feature dimension. Under the equations reported here, the resulting vector *h* is a sequence-conditioned aggregation of the functional and structural representations:

(6) $$h=\mathrm{concat}\left(y_{\mathrm{ipr}},y_{\mathrm{stru}}\right)\in R^{1\times 2560}$$

#### 4.3.2. Global Representation Learning with a Transformer Encoder

The joint representation *h* is mapped to the model space by a linear embedding layer, combined with positional encoding, and processed by a transformer encoder. Mean pooling produces the representation $h_{\mathrm{pool}}$:

(7) $$\hat{z}=\left(hW_{emb}+b_{emb}\right)+\mathrm{PE}\left(\right)$$

(8) $$h_{\mathrm{pool}}=\mathrm{MeanPool}\left(\mathrm{TransformerEncoder}\left(\hat{z}\right)\right)$$

This global representation $h_{pool}$ is sequentially passed through a linear projection, Dropout regularization, and a ReLU activation function to obtain the hidden representation $u_{1}$. Finally, a linear classifier is then used to produce logits for the five coarse-grained classes, and a softmax function is applied to generate the first-stage prediction distribution, denoted as $p_{1}$:

(9) $$u_{1}=\sigma \left(\mathrm{Dropout}\left(h_{\mathrm{pool}}W_{c1}+b_{c1}\right)\right)\in R^{64}$$

(10) $${\mathrm{output}}_{1}=u_{1}W_{c2}+b_{2}\in R^{5}$$

(11) $$p_{1}=\mathrm{softmax}\left({\mathrm{output}}_{1}\right)$$

### 4.4. Task-Specific Prediction Heads

#### 4.4.1. Hierarchical Autoregressive Classification of Ion Channels and Transporters

IonFusion adopts a hierarchical autoregressive strategy to generate the final classification probabilities p(y). Unlike conventional flat classification methods, this strategy decomposes the overall prediction probability into a coarse-grained probability and a conditional fine-grained probability according to the chain rule:

(12) p(y)=p(c|h)·p(s|c,h)

where *c* denotes the coarse-grained class and *s* denotes the fine-grained class conditioned on *c*. To condition fine-grained classification on the coarse-grained prediction, the model concatenates the global semantic representation $h_{\mathrm{pool}}$, hidden representation $u_{1}$, and coarse-grained output ${\mathrm{output}}_{1}$. The resulting conditional fused representation, denoted by fused, combines global semantics, intermediate coarse-grained features, and coarse-grained class prediction information:

(13) $$\mathrm{fused}=\mathrm{concat}(h_{\mathrm{pool}},u_{1},{\mathrm{output}}_{1})$$

(14) $$g_{1}=\sigma \left(\mathrm{Dropout}\left(\mathrm{fused}W_{f1}+b_{f1}\right)\right)\in R^{64}$$

(15) $${\mathrm{output}}_{2}=g_{1}W_{f2}+b_{f2}$$

(16) $$p_{2}=\mathrm{softmax}\left({\mathrm{output}}_{2}\right)$$

This two-stage prediction mechanism follows the predefined hierarchy for classifying ion channels and transporters. It first predicts a coarse-grained class and then conditions fine-grained class prediction on the coarse-grained representation and output. The formulation is intended to help distinguish fine-grained classes with overlapping features.

#### 4.4.2. GO Multi-Label Prediction

Unlike the single-label multiclass task of classifying ion channels and transporters, Gene Ontology (GO) term prediction is a multi-label task in which each protein may be associated with multiple GO terms. After multimodal feature fusion, IonFusion uses a separate GO prediction head to estimate the probability that each candidate GO term is associated with the input protein.

To prevent information leakage from the validation and test sets, the candidate GO term space includes only terms observed in the training set. The resulting set of retained training-set GO terms is defined as:

(17) $$\vartheta =\{g_{1},g_{2},\ldots ,g_{j},\ldots ,g_{M}\}$$

where *M* denotes the size of the GO label pool in the training set. For the *i*-th protein, its ground-truth GO annotations are encoded as an *M*-dimensional multi-hot vector:

(18) $$Y_{i}=\left[y_{i,1},y_{i,2},\ldots ,y_{i,j},\ldots ,y_{i,M}\right]\in {\left\{0,1\right\}}^{M}$$

(19) $$y_{i,j}=\left\{\begin{array}{cc} 1, & \mathrm{if}\;\mathrm{protein}\;i\;\mathrm{is}\;\mathrm{annotated}\;\mathrm{with}\;g_{j}\\ 0, & \mathrm{otherwise} \end{array}\right.$$

GO terms present in the validation or test set but absent from the training-derived label set ϑ are treated as out-of-vocabulary (OOV) ground-truth terms, denoted by $T^{\mathrm{oov}}$. Because the model output vocabulary is restricted to ϑ, these terms cannot be predicted but remain in the recall denominator to prevent recall overestimation. $T^{\mathrm{in}}$ denotes the set of in-vocabulary ground-truth GO terms contained in ϑ.

The global semantic representation $h_{\mathrm{pool}}$ generated by the transformer encoder serves as the fused feature vector for GO term prediction. A two-layer feed-forward network maps this vector into the GO term space, after which an element-wise sigmoid independently estimates the probability of each term:

(20) $${\tilde{u}}_{i}=\mathrm{Dropout}\left(\mathrm{ReLU}(h_{\mathrm{pool}}W_{1}+b_{1})\right)$$

(21) $${\hat{p}}_{i}^{\mathrm{IonFusion}}=\delta \left({\tilde{u}}_{i}W_{2}+b_{2}\right)$$

where ${\hat{p}}_{i}^{\mathrm{IonFusion}}\in \left[0,1\right]$ denotes the predicted probability that protein *i* has the *j*-th GO label $g_{j}$. Considering that GO label distributions usually exhibit a pronounced long-tail pattern, where a small number of high-frequency GO terms are shared by many proteins while a large number of low-frequency GO terms appear in only a few proteins, IonFusion therefore uses a class-weighted binary cross-entropy loss during training. The positive-class weight for GO term $g_{j}$ is defined as:

(22) $$\omega_{j}=\frac{N-n_{j}}{n_{j}}$$

where *N* denotes the total number of training samples, and $n_{j}$ denotes the number of proteins annotated with GO label $g_{j}$ in the training set. This weighting strategy increases the contribution of low-frequency GO terms to the training loss and alleviates majority-class bias caused by label imbalance. The overall GO prediction loss, averaged over all *N* training proteins and *M* GO labels, is defined as:

(23) $$L_{GO}=-\frac{1}{NM}\sum_{i=1}^{N}\sum_{j=1}^{M}[\omega_{j}y_{i,j}\log\left({\hat{p}}_{i,j}^{\mathrm{IonFusion}}\right)+\left(1-y_{i,j}\right)\log\left(1-{\hat{p}}_{i,j}^{\mathrm{IonFusion}}\right)]$$

Based on the model-predicted probabilities, this study further incorporates DIAMOND homology-search results through weighted fusion:

(24) $${\hat{P}}_{i}^{\mathrm{fusion}}=\alpha {\hat{P}}_{i}^{\mathrm{IonFusion}}+\left(1-\alpha \right){\hat{P}}_{i}^{\mathrm{diamond}}$$

During inference, the model first outputs the predicted probability ${\hat{p}}_{i,j}^{\mathrm{IonFusion}}$ of each GO label. Then, according to the optimal threshold γ and optimal fusion coefficient α selected on the validation set, the probabilities are converted into binary prediction labels. For the detailed optimal fusion coefficient α, please refer to Supplementary Table S4:

(25) $${\hat{y}}_{i,j}^{\mathrm{bin}}=\left\{\begin{array}{cc} 1, & {\hat{P}}_{i,j}^{\mathrm{fusion}}\geq \gamma \\ 0, & {\hat{P}}_{i,j}^{\mathrm{fusion}}<\gamma \end{array}\right.$$

Finally, the predicted GO label set for the *i*-th protein is defined as:

(26) $${\hat{\vartheta }}_{i}=\left\{g_{j}|{\hat{P}}_{i,j}^{\mathrm{fusion}}\geq \gamma ,g_{j}\in \vartheta \right\}$$

### 4.5. Model Implementation and Hyperparameter Settings

Models were trained with a batch size of 64. The transformer encoder used a hidden dimension of 512, eight attention heads, four encoder layers, and a dropout rate of 0.1. Model parameters were optimized using AdamW [51], with an initial learning rate of $1\times 10^{-4}$ and a weight decay of 0.01. Cross-entropy loss was used for ion-selectivity classification, whereas the class-weighted binary cross-entropy loss described above was used for multi-label GO term prediction. Automatic mixed precision and gradient clipping, with a maximum gradient norm of 1.0, were used to improve computational efficiency and training stability. The released implementation uses Python 3.12.8, NumPy 1.26.4, and PyTorch 2.5.1. It initializes the Python, NumPy, and PyTorch random-number generators through a common seed parameter and provides environment files with software versions. All experiments were run on four NVIDIA RTX A6000 GPUs.

### 4.6. Performance Metrics

Task-specific metrics were used to evaluate IonFusion. For nine-class ion-selectivity classification, overall accuracy was calculated as the proportion of correctly classified test proteins. Precision, recall, and F1 score were calculated separately for each class and then averaged with equal class weights. Macro-F1 was therefore the arithmetic mean of the nine class-specific F1 scores, not the harmonic mean of macro-precision and macro-recall. We additionally calculated support-weighted precision, recall, and F1 score as $\sum_{c}n_{c}{\mathrm{Metric}}_{c}/\sum_{c}n_{c}$, where $n_{c}$ is the evaluable test support for that method in class *c*. Given the class imbalance, macro-F1 and macro-recall were treated as the primary measures of between-class balance, whereas the weighted averages and overall accuracy summarize performance under the observed test distribution.

For multi-label GO term prediction, performance was evaluated using $F_{\max}$, AUPR, ICAUPR, and DPAUPR [52,53,54,55]. $F_{\max}$ is the maximum protein-centric F1 score across prediction thresholds, whereas AUPR summarizes performance over the precision–recall curve. ICAUPR and DPAUPR are supplementary weighted measures that emphasize information-rich and hierarchically deep GO terms, respectively. In the CAFA information-theoretic framework, term-level information accretion is defined as $-\log_{2}\mathrm{Pr}\left(g|\mathrm{Pa}\left(g\right)\right)$ [53]. In our implementation, the weight was estimated from propagated training annotations using a single-parent approximation, as detailed in Supplementary Note S1. For a GO term with one or more directly connected is_a parents, the released implementation uses the least frequent observed direct parent as the reference count, giving $\tilde{p}\left(g\right)=n_{g}/n_{p^{*}\left(g\right)}$ and $\mathrm{IC}\left(g\right)=\log_{2}\left(n_{p^{*}\left(g\right)}/n_{g}\right)$. Within each threshold–ontology setting, every evaluated method used the same train-derived vocabulary and OOV definition. Ground-truth terms absent from that vocabulary were retained in the recall and weighted-recall denominators. Detailed definitions and mathematical formulations are provided in Supplementary Note S1.

## 5. Conclusions

In summary, we developed IonFusion as a multimodal framework for the classification and GO annotation of ion channels and transporters. IonFusion uses sequence-conditioned cross-attention to combine sequence, functional-domain, and structure-aware representations. Its coarse-to-fine autoregressive classifier links five coarse-grained classes with nine fine-grained classes. For GO annotation, the framework further combines multimodal predictions with DIAMOND homology scores.

On the evaluated classification test set, IonFusion achieved a macro-F1 of 0.860, higher than that of all eight evaluated baselines. In the GO benchmark, it obtained the highest Fmax in all nine comparisons across three independently constructed sequence-identity datasets and the BPO, CCO, and MFO namespaces. Ablation experiments indicated that the three feature modalities and both classification components contributed to performance under the evaluated settings. Together, these results support the use of integrated protein representations and hierarchical prediction for the tasks examined in this study.

## Data Availability

The datasets and model files generated and analyzed during the current study are openly available in the Hugging Face repository at DOI: https://doi.org/10.57967/hf/10587. The source code, computational environment configuration, random seed settings, plotting scripts, and associated documentation are publicly available at https://github.com/maroonlove/IonFusion (accessed on 11 October 2026).

## Supplementary Materials

The following supporting information can be downloaded at: https://www.mdpi.com/article/10.3390/molecules31203679/s1. Supplementary Note S1: Evaluation Metrics. Figure S1. Predefined coarse-to-fine label hierarchy for classification of ion channels and transporters. The five coarse-grained classes (labels 0–4) are ion transporter, potassium channel, calcium channel, sodium channel, and chloride channel. The fine-grained scheme retains labels 0–4 and adds labels 5–8 for Cys-loop receptor channel, ionotropic glutamate channel, ATP-gated channel, and proton-gated channel. The connecting lines identify each added class’s parent coarse-grained class and do not represent learned biological relationships. Figure S2. Area under the protein-centric precision–recall curve (AUPR) for GO prediction. Columns correspond to Biological Process (BPO), Cellular Component (CCO), and Molecular Function (MFO). Panels (a), (b), and (c) show separately constructed datasets with sequence-identity thresholds of ≤10%, ≤30%, and ≤50%, respectively. Higher values indicate better performance within a panel. Figure S3. Information-content-weighted AUPR (ICAUPR) for GO prediction. Columns correspond to BPO, CCO, and MFO. Panels (a), (b), and (c) show separately constructed datasets with sequence-identity thresholds of ≤10%, ≤30%, and ≤50%, respectively. Information content is estimated from propagated training annotations as defined in Supplementary Note S1. Higher values indicate better performance within a panel. Figure S4. Depth- and information-content-weighted AUPR (DPAUPR) for GO prediction. Columns correspond to BPO, CCO, and MFO. Panels (a), (b), and (c) show separately constructed datasets with sequence-identity thresholds of ≤10%, ≤30%, and ≤50%, respectively. The weighting scheme is defined in Supplementary Note S1. Higher values indicate better performance within a panel. Figure S5. Distribution of label-wise AUPR values for GO terms with ontology depth >6. Columns represent BPO, CCO, and MFO, whereas rows (a), (b), and (c) show separately constructed datasets with sequence-identity thresholds of ≤10%, ≤30%, and ≤50%, respectively. Each plotted observation represents one eligible GO term. Figure S6. Performance across the three sequence-identity settings. Panels (a–d) correspond to Fmax, AUPR, ICAUPR, and DPAUPR, and columns represent BPO, CCO, and MFO. Because the threshold-specific datasets contain different proteins and GO-term compositions, the connected values summarize separate evaluation sets and should not be interpreted as repeated measurements on an identical cohort. Figure S7. Classification results for the full IonFusion model (left) and the variant without hierarchical classification (NoLayer; right) on the same evaluable test proteins used in Supplementary Table S7. In each confusion matrix, rows are true classes, columns are predicted classes, cell labels give protein counts, and the blue color scale is logarithmic. The upper bars show class-wise precision and the bars at right show class-wise recall; yellow marks values higher for IonFusion than for NoLayer. The nine classes, in axis order, are ion transporter, potassium channel, calcium channel, sodium channel, chloride channel, Cys-loop receptor channel, ionotropic glutamate channel, ATP-gated channel, and proton-gated channel. Figure S8. Motif-associated attention analysis in the potassium-channel test set. The six five-residue motifs analyzed here were selected as the six most frequent highest-attention windows among the 7515 correctly classified potassium-channel proteins, as reported in Supplement Table S10, and were subsequently searched across the entire potassium-channel test set. If the same motif occurred multiple times within a protein, only the occurrence with the highest attention-derived score was retained for score calculation. The upper panel shows the number of proteins containing each motif, with the most frequently observed motif highlighted in yellow and the remaining motifs shown in blue. The lower panel shows the mean attention-derived score of each motif across motif-containing proteins, with the motif exhibiting the highest mean score highlighted in yellow. Figure S9. Change in the potassium-channel model score after perturbation of the TVGYG motif. Among the 7515 correctly classified potassium-channel proteins in the test set, 2553 contained an exact TVGYG motif and were included in this perturbation analysis. For the sequence modality, the residues within the TVGYG segment were randomly mutated to other amino acids. For the functional-domain and structural modalities, the embedding components corresponding to the TVGYG region were replaced with random values sampled from the interval [−5, 5]. Each point represents one protein, and the vertical axis indicates the potassium-channel model score after perturbation minus that before perturbation; the dashed horizontal line denotes no change. Across the 2553 proteins, the potassium-channel score decreased for 2070 (81.08%), increased for 360 (14.10%), and remained unchanged for 123 (4.82%). The left group consists of proteins whose highest-attention five-residue window was TVGYG (n=1710), whereas the right group consists of the remaining TVGYG-containing proteins (n=843). In the left group, 1090 proteins (63.74%) were still predicted as potassium channels after perturbation, whereas 620 proteins (36.26%) were no longer predicted as potassium channels and were instead assigned to one of the other eight protein classes. In the right group, 670 proteins (79.48%) remained predicted as potassium channels, whereas 173 proteins (20.52%) were reassigned to one of the other eight classes. Blue points denote proteins that remained classified as potassium channels after perturbation, whereas orange points denote proteins whose predicted class changed from potassium channel to one of the other eight classes. Table S1. Composition of the original ion channel and transporter dataset before quality control. Table S2. Distributions of the training and test sets used for model evaluation. Labels 0–8 correspond to ion transporter, potassium channel, calcium channel, sodium channel, chloride channel, Cys-loop receptor channel, ionotropic glutamate channel, ATP-gated channel, and proton-gated channel, respectively. During quality control, 94 records labeled as Null and 1605 records with abnormal sequence lengths, incomplete annotations, or apparent annotation errors were removed, resulting in a curated dataset of 249,183 proteins. Subsequently, 10,612 training proteins and 2831 test proteins were excluded because sequence, structure, and text embeddings were not simultaneously available for the same protein. The final training and test sets therefore comprised 211,660 and 24,080 proteins, respectively. Table S3. GO dataset statistics across sequence-identity thresholds. PID is the number of unique protein identifiers, and GO is the number of unique propagated GO terms in each split. Threshold-specific datasets have equal split sizes but different protein and GO-term compositions. Table S4. Best DIAMOND alpha parameters across different sequence identity thresholds. Table S5. Aggregate classification performance of IonFusion and the evaluated methods. Macro averages assign equal weight to all nine classes, whereas weighted averages are calculated according to the class-specific support of the evaluable test samples for each method. All values are reported to three decimal places. Table S6. (A) Class-wise precision of IonFusion and the evaluated methods across the nine classification categories. Labels 0–8 denote ion transporter, potassium channel, calcium channel, sodium channel, chloride channel, Cys-loop receptor channel, ionotropic glutamate channel, ATP-gated channel, and proton-gated channel, respectively. (B) Class-wise recall of IonFusion and the evaluated methods across the nine classification categories. (C) Class-wise F1-score of IonFusion and the evaluated methods across the nine classification categories. Table S7. Ablation performance of IonFusion variants. Table S8. (A) Performance comparison under different sequence identity thresholds: Sequence identity ≤10%. (B) Performance comparison under different sequence identity thresholds (continued): Sequence identity ≤30%. (C) Performance comparison under different sequence identity thresholds (continued): Sequence identity ≤50%. Table S9. Out-of-vocabulary GO annotations in the test sets. The prediction vocabulary contains unique GO terms observed in the corresponding training split. OOV annotations are test annotation occurrences whose GO terms are absent from that vocabulary. Affected proteins have at least one OOV annotation. Percentages use the total number of propagated test annotation occurrences or test proteins as the denominator. Within each threshold–ontology setting, the vocabulary and OOV set are method-independent; all methods were evaluated against the same propagated ground truth with OOV terms retained in the recall denominators. Table S10. The six most frequent highest-attention five-residue windows among 7515 correctly classified potassium-channel proteins. Each protein contributes one five-residue window with the highest attention-derived score, and the first six rows list the most frequent individual sequences ranked by occurrence. In addition, the final row reports the cumulative number and proportion of proteins whose highest-attention window is either exact TVGYG or differs from TVGYG by one substitution at positions 1–4, with the fifth residue constrained to G; this cumulative count includes exact TVGYG. These statistics refer only to the highest-attention window of each protein rather than motif occurrence anywhere in the full sequence. Table S11. Occurrence of TVGYG-like sequences across the nine fine-grained classes in the final embedding-complete classification test set (n= 24,080; class denominators are reported in Supplementary Table S2). Proteins were grouped according to their true class labels and counted at most once under each criterion, regardless of the number of motif occurrences. The TVGYG-like criterion includes exact TVGYG and five-residue windows differing at one of positions 1–4; the fifth residue must remain G. This position-restricted rule reflects the strong functional constraint on the terminal glycine observed in KcsA mutagenesis, as cited in the main text. Percentages were calculated relative to the total number of proteins in each fine-grained class. Motif occurrence was assessed across the full-length sequence for all analyzed test proteins, regardless of prediction correctness. Table S12. IonFusion performance on three nested test subsets derived from the original predict_50 test set for biological process (BPO), cellular component (CCO), and molecular function (MFO). The original dataset was constructed using a 50% CD-HIT sequence-identity threshold. We then used MMseqs2 to calculate the maximum sequence identity of each test protein to the training set and selected proteins with maximum identities below 10% and 30% to form the 10% and 30% subsets, respectively. The 50% group represents the complete original test set.
