# Supplementary material for: IonFusion: Multimodal Fusion for Hierarchical Classification and Functional Annotation of Ion Channels and Transporters
Source: Molecules. 2026 Oct 14;31(20):3679. doi: 10.3390/molecules31203679 (PMC13707812; doi:10.3390/molecules31203679)
Supplement: Supplementary file 1 [file molecules-31-03679-s001.zip › molecules-4566715-supplementary.pdf]

Supplementary Materials: IonFusion: Multimodal Fusion for Hierarchical Classification and Functional Annotation of Ion Channels and Transporters

Ruiqing Zheng, Wentao Yu, Qiurong Yang, Zelin Zhao, Wenxuan He, Shubiao Qu, Taozhe Li and Wenkang Wang

1. Supplementary Note S1: Evaluation Metrics

Reference numbers in this supplementary material follow the References section of the main article.

1.1. Classification of Ion Channels and Transporters

For the nine-class classification task, we evaluate performance using overall accuracy and macro-averaged precision, recall, and F1 score. Overall accuracy is the proportion of test proteins assigned to the correct class:

$$\text{Accuracy} = \frac{1}{N}\sum_{i=1}^{N}\mathbb{I}(y_i = \hat{y}_i), \tag{S1}$$

where N is the number of test proteins. For each class c, precision, recall, and F1 score are calculated in a one-versus-rest manner:

$$\text{Precision}_c = \frac{TP_c}{TP_c + FP_c} \tag{S2}$$

$$\text{Recall}_c = \frac{TP_c}{TP_c + FN_c} \tag{S3}$$

$$\text{F1}_c = 2 \times \frac{\text{Precision}_c \times \text{Recall}_c}{\text{Precision}_c + \text{Recall}_c}. \tag{S4}$$

The macro-averaged metrics assign equal weight to all nine classes:

$$\text{Macro-Metric} = \frac{1}{9}\sum_{c=0}^{8}\text{Metric}_c, \tag{S5}$$

where Metric denotes precision, recall, or F1. Macro-F1 is the arithmetic mean of the nine class-specific F1 scores; it is not calculated as the harmonic mean of macro-precision and macro-recall. Macro-averaged results are reported in Supplementary Table S5, and the corresponding class-wise results are reported in Supplementary Table S6.

Given the substantial class imbalance in the nine-class test set, overall accuracy alone can be dominated by the majority classes and therefore does not fully characterize model performance across all fine-grained classes. We therefore place greater emphasis on macro-averaged and class-wise metrics, which give equal consideration to each class and more directly reflect performance on underrepresented classes. For completeness, we additionally report support-weighted averages to summarize performance under the observed test-set distribution:

$$\text{Weighted-Metric} = \frac{1}{N}\sum_{c=0}^{8} n_c\text{Metric}_c, \tag{S6}$$

For each method, n_c denotes its evaluable test support in class c and $N = \sum_c n_c$. Macro- and weighted-average metrics are reported together in Supplementary Table S5. The weighted averages and overall accuracy describe performance under the empirical class distribution,

whereas the macro-averaged and class-wise results provide a more balanced assessment across classes.

1.2. GO Function Annotation

For GO label prediction, this study uses Fmax, AUPR, ICAUPR, and DPAUPR to evaluate model performance [47,49]. Fmax and AUPR are the primary protein-centric measures. ICAUPR and DPAUPR are supplementary weighted measures. At threshold γ, precision and recall are defined as:

$$\mathrm{Pr}(\gamma) = \frac{1}{N_\gamma} \sum_{i:|\hat{\vartheta}_i(\gamma)|>0} \frac{|\hat{\vartheta}_i(\gamma) \cap \mathrm{T}_i^{in}|}{|\hat{\vartheta}_i(\gamma)|} \tag{S7}$$

$$\mathrm{Rc}(\gamma) = \frac{1}{N} \sum_{i=1}^{N} \frac{|\hat{\vartheta}_i(\gamma) \cap \mathrm{T}_i^{in}|}{|\mathrm{T}_i^{in}| + |\mathrm{T}_i^{oov}|} \tag{S8}$$

$$\mathrm{Fmax} = \max_{\gamma} \left\{ \frac{2\mathrm{Pr}(\gamma)\mathrm{Rc}(\gamma)}{\mathrm{Pr}(\gamma) + \mathrm{Rc}(\gamma)} \right\} \tag{S9}$$

Fmax is the maximum F1 score over all thresholds γ. N_γ denotes the number of proteins for which at least one GO label is predicted at threshold γ, and N denotes the total number of test proteins. The prediction vocabulary contains only GO terms observed in the training split. A validation or test term outside that vocabulary is designated an OOV ground-truth term. Such terms cannot be predicted, but they remain in the recall denominator. This conservative treatment prevents the exclusion of unpredicted OOV annotations from inflating recall. The same OOV annotations are retained in the denominators of the information-content- and depth-weighted recall measures below. Within a given sequence-identity threshold and ontology, the vocabulary, propagated ground-truth annotations, and resulting OOV set are fixed by the data split rather than by the prediction method; all methods in Supplementary Table S8 were therefore evaluated with the same OOV treatment.

AUPR denotes the area under the precision-recall curve, which is used to measure the overall predictive ability of the model under long-tailed and imbalanced GO label distributions.

To describe performance on information-rich and hierarchically deep GO terms, this study additionally reports ICAUPR and DPAUPR. Let $\mathrm{IC}(g)$ denote the information content of GO label g, and let $\mathrm{DP}(g)$ denote its depth weight. At threshold γ, IC-weighted precision and recall are defined as:

$$\mathrm{icPr}(\gamma) = \frac{1}{N_\gamma} \sum_{i:|\hat{\vartheta}_i(\gamma)|>0} \frac{\sum_{g\in\hat{\vartheta}_i(\gamma)\cap\mathrm{T}_i^{in}} \mathrm{IC}(g)}{\sum_{g\in\hat{\vartheta}_i(\gamma)} \mathrm{IC}(g)} \tag{S10}$$

$$\mathrm{icRc}(\gamma) = \frac{1}{N} \sum_{i=1}^{N} \frac{\sum_{g\in\hat{\vartheta}_i(\gamma)\cap\mathrm{T}_i^{in}} \mathrm{IC}(g)}{\sum_{g\in\mathrm{T}_i^{in}} \mathrm{IC}(g) + \sum_{g\in\mathrm{T}_i^{oov}} \mathrm{IC}(g)} \tag{S11}$$

In the CAFA information-theoretic framework, term-level information accretion is defined as $-\log_2$ of the conditional probability of observing a term given all its direct parents [47,48]:

$$\mathrm{IC}(g) = -\log_2 \mathrm{Pr}\big(g \mid \mathrm{Pa}(g)\big). \tag{S12}$$

Our implementation instead estimates a single-parent approximation from propagated training annotations. For terms with one or more directly connected `is_a` parents,

$$\widetilde{p}(g) = \frac{n_g}{n_{p^*(g)}}, \qquad p^*(g) = \arg\min_{p \in P(g)} n_p, \tag{S13}$$

and therefore

$$\mathrm{IC}(g) = \log_2\left(\frac{n_{p^*(g)}}{n_g}\right). \tag{S14}$$

Here, $P(g)$ is the set of directly connected `is_a` parents, n_g is the number of propagated training annotations containing g, and $n_{p^*(g)}$ is the smallest observed count among its direct parents. For terms with multiple direct parents, the single-parent reference count need not equal the count conditional on all parents. The reported ICAUPR and DPAUPR use this implementation-specific IC weight. For a term without an observed direct parent, the implementation sets the reference count equal to n_g, yielding $\mathrm{IC}(g) = 0$. DP-weighted precision and recall are defined as:

$$\mathrm{dpPr}(\gamma) = \frac{1}{N_\gamma} \sum_{i:|\hat{\vartheta}_i(\gamma)|>0} \frac{\sum_{g \in \hat{\vartheta}_i(\gamma) \cap \mathrm{T}_i^{in}} \mathrm{DP}(g)}{\sum_{g \in \hat{\vartheta}_i(\gamma)} \mathrm{DP}(g)} \tag{S15}$$

$$\mathrm{dpRc}(\gamma) = \frac{1}{N} \sum_{i=1}^{N} \frac{\sum_{g \in \hat{\vartheta}_i(\gamma) \cap \mathrm{T}_i^{in}} \mathrm{DP}(g)}{\sum_{g \in \mathrm{T}_i^{in}} \mathrm{DP}(g) + \sum_{g \in \mathrm{T}_i^{oov}} \mathrm{DP}(g)} \tag{S16}$$

$$\mathrm{DP}(g) = \log_2(\max(\mathrm{depth}(g), 1)) \cdot \mathrm{IC}(g) \tag{S17}$$

where depth(g) is the ontology depth returned by the released implementation. All probability thresholds are evaluated on the same 101-point grid from 0 to 1, and the threshold maximizing F1 is used for Fmax. In a tie, the threshold with the smaller semantic-distance score is selected by the implementation.

2. Supplementary Figures

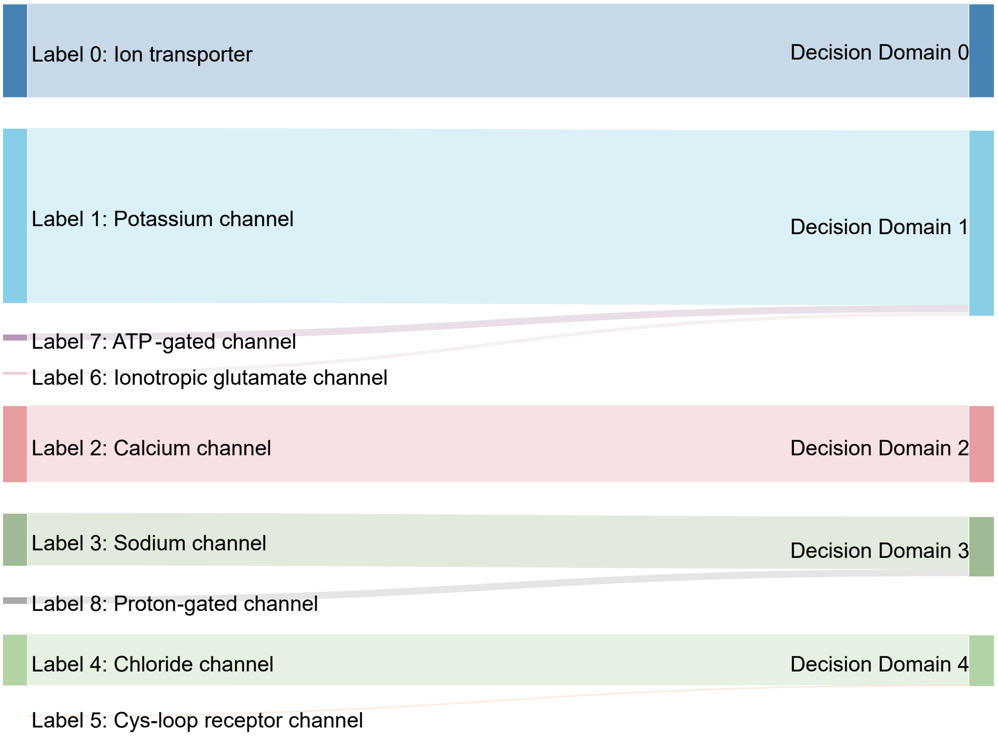

Figure S1. Predefined coarse-to-fine label hierarchy for classification of ion channels and transporters. The five coarse-grained classes (labels 0–4) are ion transporter, potassium channel, calcium channel, sodium channel, and chloride channel. The fine-grained scheme retains labels 0–4 and adds labels 5–8 for Cys-loop receptor channel, ionotropic glutamate channel, ATP-gated channel, and proton-gated channel. The connecting lines identify each added class's parent coarse-grained class and do not represent learned biological relationships.

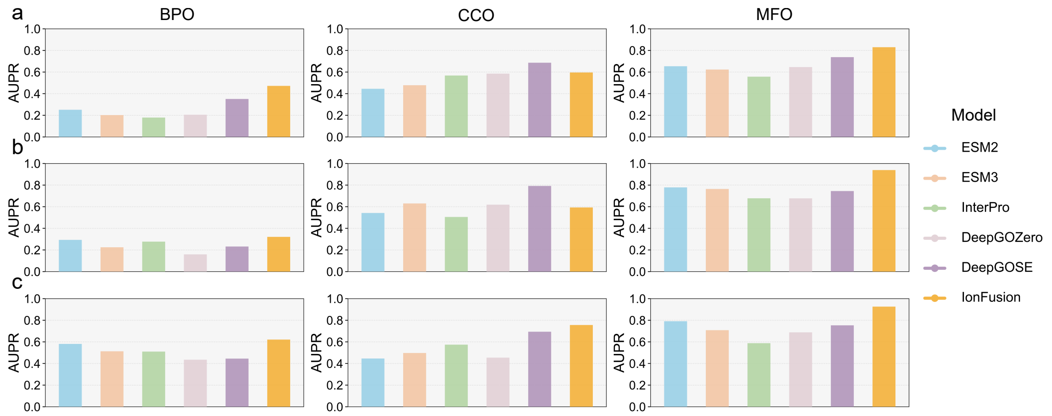

Figure S2. Area under the protein-centric precision–recall curve (AUPR) for GO prediction. Columns correspond to Biological Process (BPO), Cellular Component (CCO), and Molecular Function (MFO). Panels (a), (b), and (c) show separately constructed datasets with sequence-identity thresholds of $\leq$ 10%, $\leq$ 30%, and $\leq$ 50%, respectively. Higher values indicate better performance within a panel.

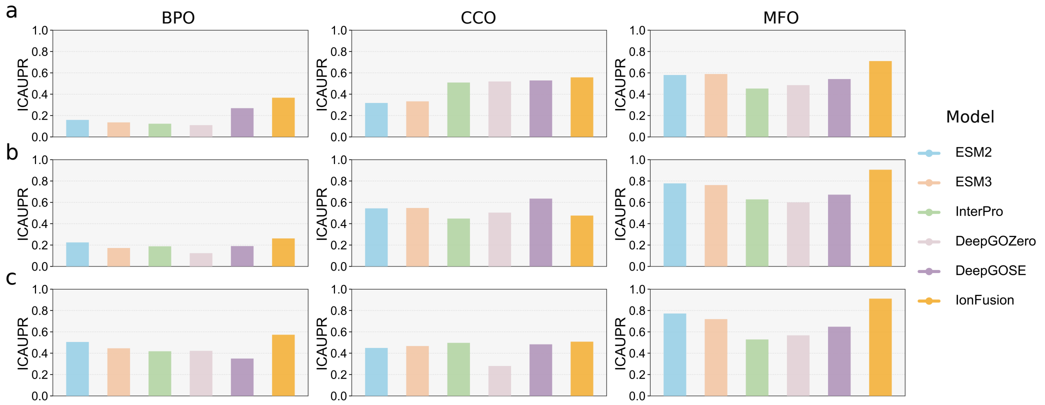

Figure S3. Information-content-weighted AUPR (ICAUPR) for GO prediction. Columns correspond to BPO, CCO, and MFO. Panels (a), (b), and (c) show separately constructed datasets with sequence-identity thresholds of $\leq$ 10%, $\leq$ 30%, and $\leq$ 50%, respectively. Information content is estimated from propagated training annotations as defined in Supplementary Note S1. Higher values indicate better performance within a panel.

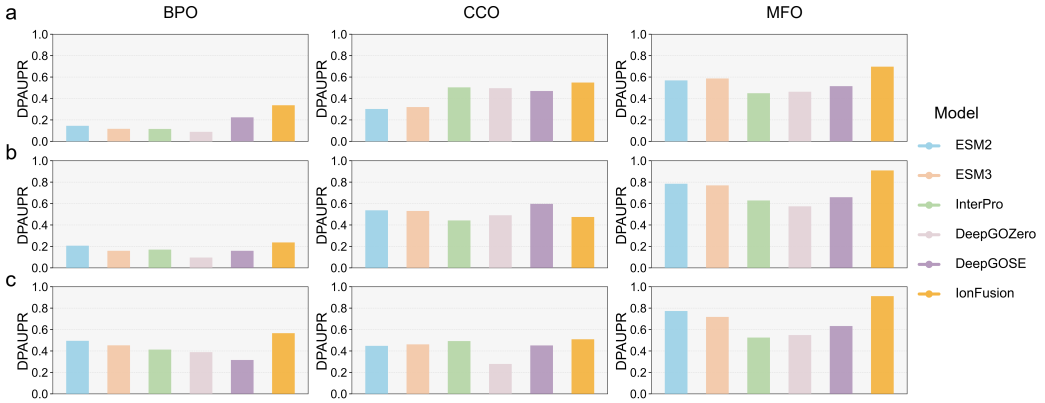

Figure S4. Depth- and information-content-weighted AUPR (DPAUPR) for GO prediction. Columns correspond to BPO, CCO, and MFO. Panels (a), (b), and (c) show separately constructed datasets with sequence-identity thresholds of $\leq$ 10%, $\leq$ 30%, and $\leq$ 50%, respectively. The weighting scheme is defined in Supplementary Note S1. Higher values indicate better performance within a panel.

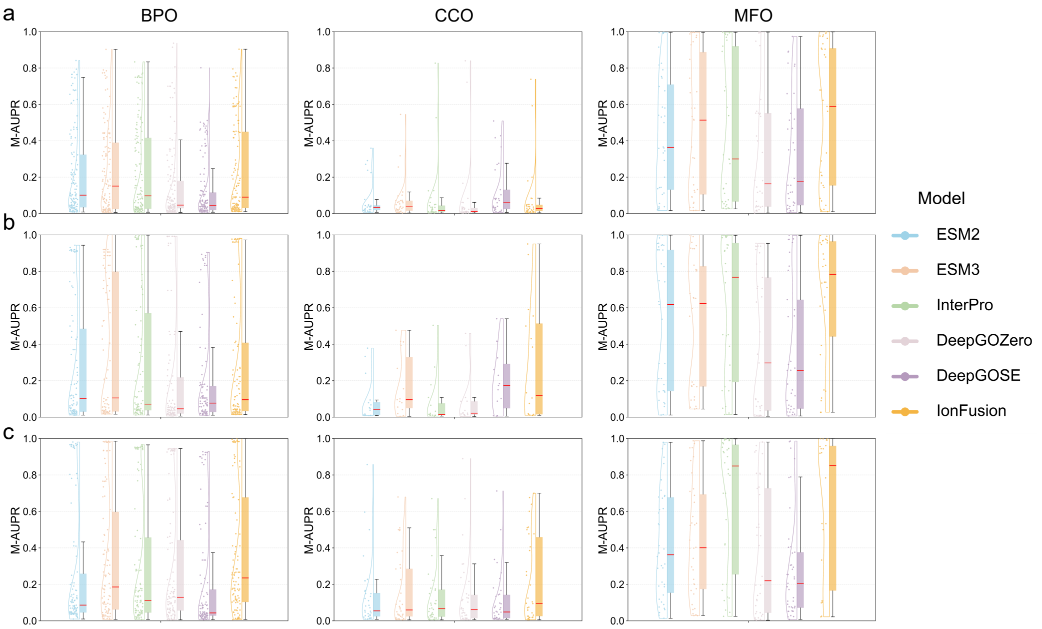

Figure S5. Distribution of label-wise AUPR values for GO terms with ontology depth > 6. Columns represent BPO, CCO, and MFO, whereas rows (a), (b), and (c) show separately constructed datasets with sequence-identity thresholds of $\leq$ 10%, $\leq$ 30%, and $\leq$ 50%, respectively. Each plotted observation represents one eligible GO term.

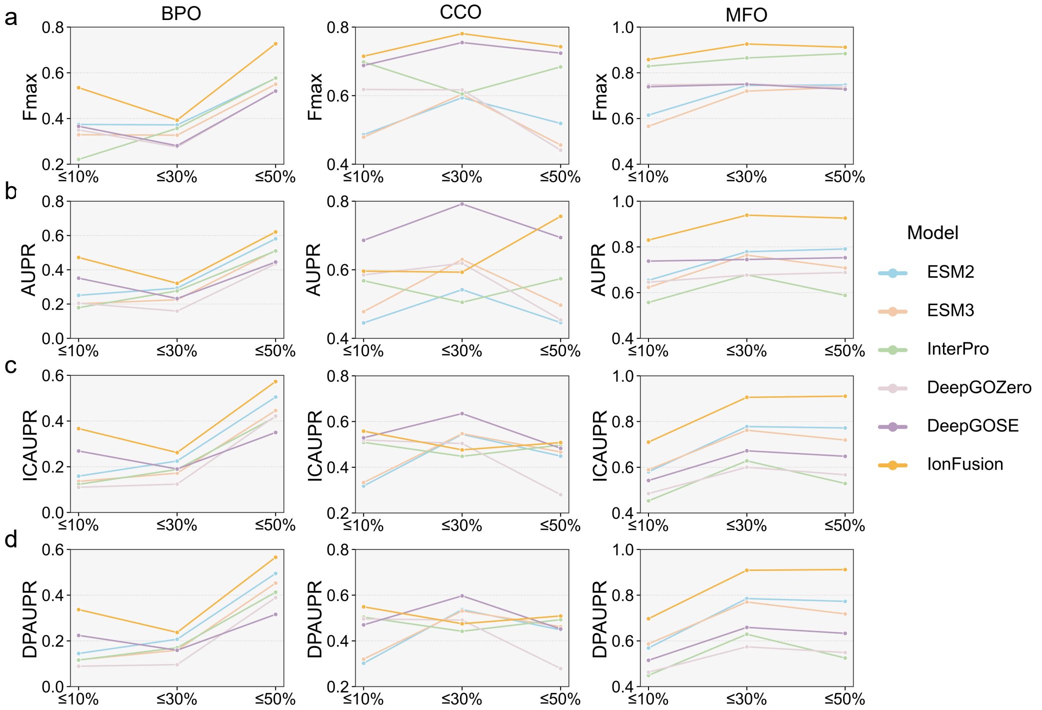

Figure S6. Performance across the three sequence-identity settings. Panels (a–d) correspond to Fmax, AUPR, ICAUPR, and DPAUPR, and columns represent BPO, CCO, and MFO. Because the threshold-specific datasets contain different proteins and GO-term compositions, the connected values summarize separate evaluation sets and should not be interpreted as repeated measurements on an identical cohort.

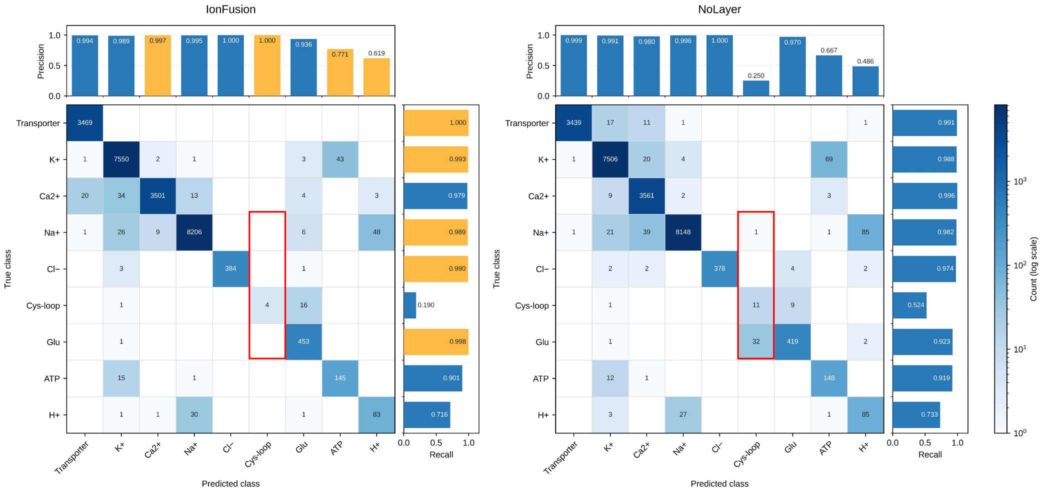

Figure S7. Classification results for the full IonFusion model (left) and the variant without hierarchical classification (NoLayer; right) on the same evaluable test proteins used in Supplementary Table S7. In each confusion matrix, rows are true classes, columns are predicted classes, cell labels give protein counts, and the blue color scale is logarithmic. The upper bars show class-wise precision and the bars at right show class-wise recall; yellow marks values higher for IonFusion than for NoLayer. The nine classes, in axis order, are ion transporter, potassium channel, calcium channel, sodium channel, chloride channel, Cys-loop receptor channel, ionotropic glutamate channel, ATP-gated channel, and proton-gated channel.

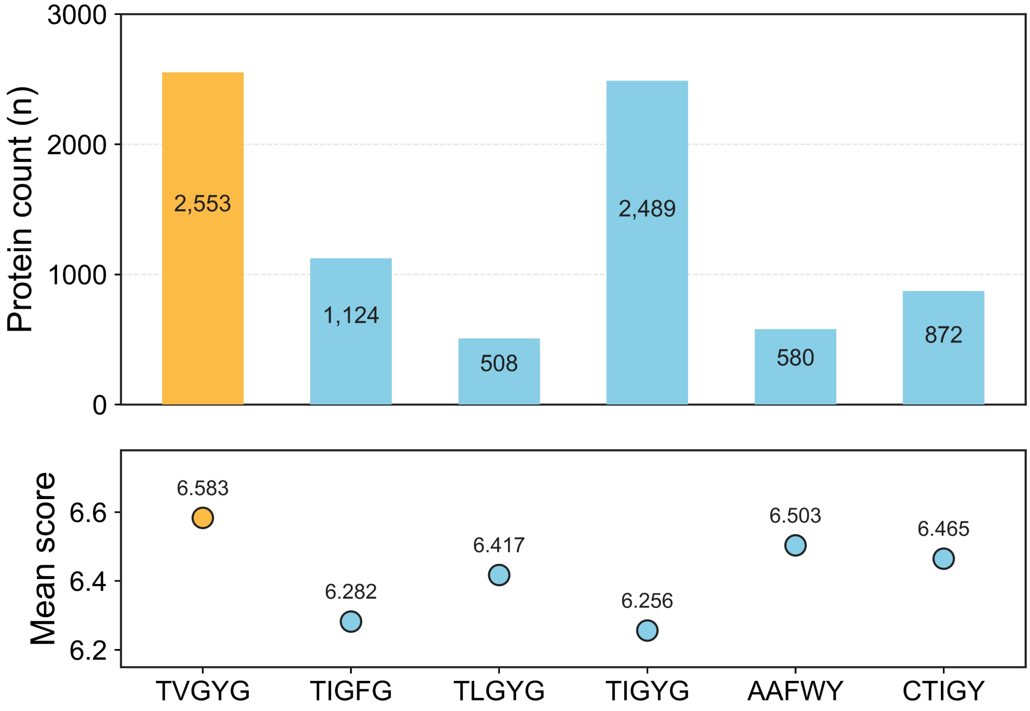

Figure S8. Motif-associated attention analysis in the potassium-channel test set. The six five-residue motifs analyzed here were selected as the six most frequent highest-attention windows among the 7,515 correctly classified potassium-channel proteins, as reported in Supplement Table S10, and were subsequently searched across the entire potassium-channel test set. If the same motif occurred multiple times within a protein, only the occurrence with the highest attention-derived score was retained for score calculation. The upper panel shows the number of proteins containing each motif, with the most frequently observed motif highlighted in yellow and the remaining motifs shown in blue. The lower panel shows the mean attention-derived score of each motif across motif-containing proteins, with the motif exhibiting the highest mean score highlighted in yellow.

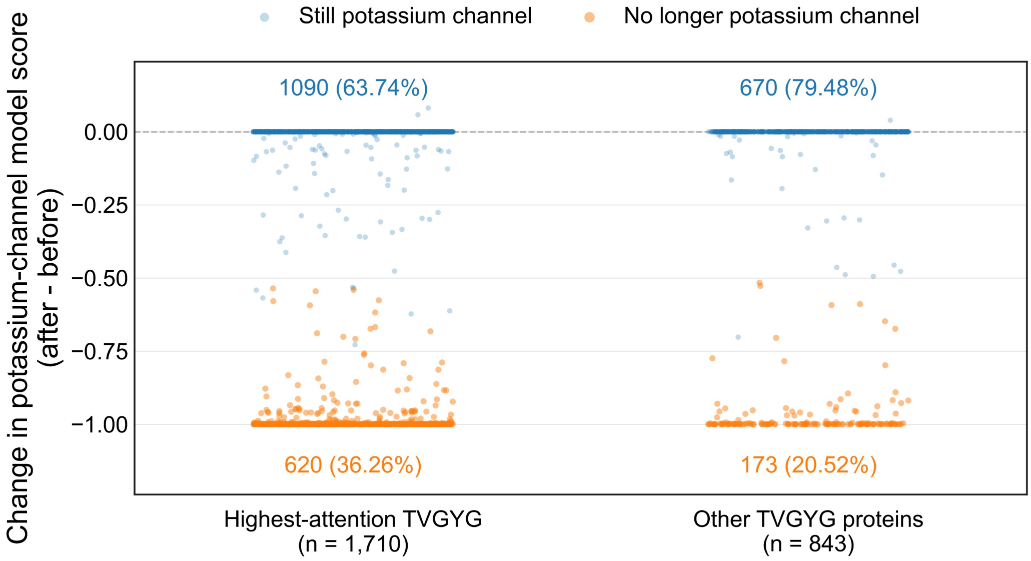

Figure S9. Change in the potassium-channel model score after perturbation of the `TVGYG` motif. Among the 7,515 correctly classified potassium-channel proteins in the test set, 2,553 contained an exact `TVGYG` motif and were included in this perturbation analysis. For the sequence modality, the residues within the `TVGYG` segment were randomly mutated to other amino acids. For the functional-domain and structural modalities, the embedding components corresponding to the `TVGYG` region were replaced with random values sampled from the interval $[-5, 5]$. Each point represents one protein, and the vertical axis indicates the potassium-channel model score after perturbation minus that before perturbation; the dashed horizontal line denotes no change. Across the 2,553 proteins, the potassium-channel score decreased for 2,070 (81.08%), increased for 360 (14.10%), and remained unchanged for 123 (4.82%). The left group consists of proteins whose highest-attention five-residue window was `TVGYG` ($n = 1,710$), whereas the right group consists of the remaining `TVGYG`-containing proteins ($n = 843$). In the left group, 1,090 proteins (63.74%) were still predicted as potassium channels after perturbation, whereas 620 proteins (36.26%) were no longer predicted as potassium channels and were instead assigned to one of the other eight protein classes. In the right group, 670 proteins (79.48%) remained predicted as potassium channels, whereas 173 proteins (20.52%) were reassigned to one of the other eight classes. Blue points denote proteins that remained classified as potassium channels after perturbation, whereas orange points denote proteins whose predicted class changed from potassium channel to one of the other eight classes.

3. Supplementary Tables

Table S1. Composition of the original ion channel and transporter dataset before quality control.

Protein category	Label	Number
Ion transporter	0	50,000
Potassium channel	1	94,163
Calcium channel	2	41,199
Sodium channel	3	28,412
Chloride channel	4	27,539
Cys-loop receptor channel	5	121
Ionotropic glutamate channel	6	1,875
ATP-gated channel	7	3,763
Proton-gated channel	8	3,716
Null	Null	94

Table S2. Distributions of the training and test sets used for model evaluation. Labels 0–8 correspond to ion transporter, potassium channel, calcium channel, sodium channel, chloride channel, Cys-loop receptor channel, ionotropic glutamate channel, ATP-gated channel, and proton-gated channel, respectively. During quality control, 94 records labeled as Null and 1,605 records with abnormal sequence lengths, incomplete annotations, or apparent annotation errors were removed, resulting in a curated dataset of 249,183 proteins. Subsequently, 10,612 training proteins and 2,831 test proteins were excluded because sequence, structure, and text embeddings were not simultaneously available for the same protein. The final training and test sets therefore comprised 211,660 and 24,080 proteins, respectively.

Split/Label	0	1	2	3	4	5	6	7	8
Train	42,704	80,998	33,613	18,754	26,975	100	1,401	3,555	3,560
Test	3,469	7,600	3,575	8,296	388	21	454	161	116

Table S3. GO dataset statistics across sequence-identity thresholds. PID is the number of unique protein identifiers, and GO is the number of unique propagated GO terms in each split. Threshold-specific datasets have equal split sizes but different protein and GO-term compositions.

Identity	Ontology	Split	PID	GO
10%	BPO	Train	1387	2106
		Val	173	890
		Test	173	697
	CCO	Train	2440	415
		Val	305	183
		Test	305	153
	MFO	Train	2505	364
		Val	313	191
		Test	313	188
30%	BPO	Train	1387	2065
		Val	173	599
		Test	173	978
	CCO	Train	2440	411
		Val	305	141
		Test	305	183
	MFO	Train	2505	429
		Val	313	121
		Test	313	145
50%	BPO	Train	1387	2295
		Val	173	609
		Test	173	565
	CCO	Train	2440	388
		Val	305	206
		Test	305	220
	MFO	Train	2505	422
		Val	313	146
		Test	313	150

Table S4. Best DIAMOND alpha parameters across different sequence identity thresholds.

Identity	Ontology	diamond_best_alpha
10%	BPO	0.57
	CCO	0.91
	MFO	0.64
30%	BPO	0.49
	CCO	0.33
	MFO	0.89
50%	BPO	0.57
	CCO	0.02
	MFO	0.50

Table S5. Aggregate classification performance of IonFusion and the evaluated methods. Macro averages assign equal weight to all nine classes, whereas weighted averages are calculated according to the class-specific support of the evaluable test samples for each method. All values are reported to three decimal places.

Method	Accuracy	Macro average			Weighted average		
		Precision	Recall	F1-score	Precision	Recall	F1-score
**IonFusion**	**0.988**	**0.922**	**0.862**	**0.860**	**0.989**	**0.988**	**0.989**
AAC-SVM	0.897	0.768	0.678	0.695	0.904	0.897	0.899
AAC-MLP	0.887	0.671	0.659	0.652	0.895	0.887	0.890
DPC-SVM	0.869	0.696	0.629	0.621	0.883	0.869	0.872
DPC-MLP	0.943	0.837	0.720	0.721	0.948	0.943	0.944
PSI-SVM	0.907	0.849	0.689	0.715	0.921	0.907	0.908
PSI-MLP	0.942	0.714	0.718	0.711	0.943	0.942	0.942
DeepPLM	0.976	0.651	0.657	0.654	0.966	0.976	0.971
DeepIon	0.865	0.661	0.693	0.662	0.883	0.865	0.868

Table S6. (A) Class-wise precision of IonFusion and the evaluated methods across the nine classification categories. Labels 0–8 denote ion transporter, potassium channel, calcium channel, sodium channel, chloride channel, Cys-loop receptor channel, ionotropic glutamate channel, ATP-gated channel, and proton-gated channel, respectively.

Precision

Method	0	1	2	3	4	5	6	7	8
**IonFusion**	**0.994**	**0.989**	**0.997**	**0.995**	**1.000**	**1.000**	**0.936**	**0.771**	**0.619**
AAC-SVM	0.983	0.821	0.880	0.975	0.502	0.750	0.960	0.687	0.358
AAC-MLP	0.964	0.852	0.842	0.961	0.386	0.429	0.929	0.531	0.144
DPC-SVM	0.934	0.837	0.817	0.963	0.317	0.500	0.981	0.679	0.238
DPC-MLP	0.986	0.933	0.963	0.963	0.467	1.000	0.984	0.771	0.464
PSI-SVM	1.000	0.785	0.934	0.991	0.879	1.000	0.987	0.704	0.361
PSI-MLP	0.997	0.928	0.889	0.979	0.717	0.000	0.959	0.621	0.336
DeepPLM	0.998	0.951	0.984	0.986	0.997	0.000	0.942	0.000	0.000
DeepIon	0.998	0.777	0.832	0.968	0.434	0.000	0.954	0.651	0.337

Table S6. (B) Class-wise recall of IonFusion and the evaluated methods across the nine classification categories.

Recall

Method	0	1	2	3	4	5	6	7	8
**IonFusion**	**1.000**	**0.993**	**0.979**	**0.989**	**0.990**	**0.190**	**0.998**	**0.901**	**0.716**
AAC-SVM	0.934	0.927	0.863	0.895	0.721	0.143	0.802	0.568	0.250
AAC-MLP	0.931	0.880	0.878	0.905	0.680	0.143	0.808	0.586	0.121
DPC-SVM	0.869	0.908	0.813	0.884	0.816	0.095	0.910	0.235	0.129
DPC-MLP	0.944	0.922	0.943	0.983	0.913	0.143	0.963	0.562	0.112
PSI-SVM	0.950	0.990	0.893	0.845	0.841	0.050	0.808	0.469	0.351
PSI-MLP	0.975	0.962	0.958	0.920	0.890	0.000	0.938	0.395	0.423
DeepPLM	0.988	0.999	0.989	0.976	0.967	0.000	0.993	0.000	0.000
DeepIon	0.812	0.945	0.815	0.863	0.877	0.000	0.963	0.438	0.523

Table S6. (C) Class-wise F1-score of IonFusion and the evaluated methods across the nine classification categories.

	F1-score								
**Method**	**0**	**1**	**2**	**3**	**4**	**5**	**6**	**7**	**8**
**IonFusion**	**0.997**	**0.991**	**0.988**	**0.992**	**0.995**	**0.320**	**0.966**	**0.831**	**0.664**
AAC-SVM	0.958	0.871	0.872	0.933	0.592	0.240	0.874	0.622	0.294
AAC-MLP	0.947	0.866	0.859	0.932	0.493	0.214	0.865	0.557	0.131
DPC-SVM	0.900	0.871	0.815	0.922	0.457	0.160	0.944	0.349	0.168
DPC-MLP	0.965	0.927	0.953	0.973	0.618	0.250	0.973	0.650	0.181
PSI-SVM	0.974	0.876	0.913	0.912	0.860	0.095	0.889	0.563	0.356
PSI-MLP	0.986	0.945	0.922	0.949	0.794	0.000	0.949	0.483	0.375
DeepPLM	0.993	0.974	0.987	0.981	0.982	0.000	0.967	0.000	0.000
DeepIon	0.895	0.852	0.823	0.913	0.581	0.000	0.958	0.524	0.410

Table S7. Ablation performance of IonFusion variants.

Index	Class	Accuracy	Precision	Recall	F1-score
1	IonFusion	0.988	0.922	0.862	0.860
2	IPR+Str	0.983	0.760	0.768	0.756
3	Seq+Str	0.986	0.807	0.820	0.811
4	Seq+IPR	0.987	0.852	0.796	0.819
5	Seq	0.979	0.814	0.759	0.771
6	NoLayer	0.984	0.815	0.892	0.843
7	NoAR	0.984	0.817	0.774	0.792

Table S8. (A) Performance comparison under different sequence identity thresholds: Sequence identity $\leq$ 10%.

	Sequence identity $\leq$ 10%					
**Ontology**	**Method**	**Fmax**	**AUPR**	**ICAUPR**	**DPAUPR**	**thr_fmax**
BPO	IonFusion	**0.535**	**0.472**	**0.367**	**0.337**	**0.55**
	ESM2	0.374	0.251	0.159	0.145	0.55
	ESM3	0.329	0.202	0.136	0.117	0.83
	InterPro	0.221	0.179	0.123	0.116	0.61
	DeepGOZero	0.350	0.205	0.110	0.089	0.48
	DeepGOSE	0.366	0.351	0.269	0.224	0.31
CCO	IonFusion	**0.715**	**0.596**	**0.558**	**0.549**	**0.90**
	ESM2	0.486	0.445	0.318	0.302	0.30
	ESM3	0.479	0.478	0.333	0.320	0.53
	InterPro	0.698	0.568	0.509	0.504	0.11
	DeepGOZero	0.618	0.585	0.519	0.496	0.24
	DeepGOSE	0.688	0.686	0.529	0.470	0.48
MFO	IonFusion	**0.858**	**0.830**	**0.710**	**0.697**	**0.59**
	ESM2	0.615	0.654	0.580	0.569	0.63
	ESM3	0.566	0.623	0.589	0.587	0.43
	InterPro	0.829	0.557	0.453	0.449	0.94
	DeepGOZero	0.747	0.646	0.485	0.463	0.53
	DeepGOSE	0.739	0.738	0.542	0.515	0.55

Table S8. (B) Performance comparison under different sequence identity thresholds (continued): Se-quence identity ≤ 30%.

**Sequence identity** ≤ 30%						
**Ontology**	**Method**	**Fmax**	**AUPR**	**ICAUPR**	**DPAUPR**	**thr_fmax**
BPO	IonFusion	**0.393**	**0.321**	**0.262**	**0.237**	**0.47**
	ESM2	0.372	0.293	0.225	0.207	0.69
	ESM3	0.327	0.225	0.172	0.159	0.64
	InterPro	0.357	0.277	0.188	0.170	0.57
	DeepGOZero	0.275	0.159	0.124	0.096	0.41
	DeepGOSE	0.281	0.232	0.190	0.159	0.43
CCO	IonFusion	**0.781**	**0.593**	**0.476**	**0.475**	**0.32**
	ESM2	0.594	0.542	0.544	0.537	0.51
	ESM3	0.604	0.630	0.547	0.531	0.52
	InterPro	0.605	0.505	0.448	0.442	0.98
	DeepGOZero	0.617	0.619	0.504	0.491	0.20
	DeepGOSE	0.755	0.792	0.635	0.597	0.36
MFO	IonFusion	**0.926**	**0.939**	**0.906**	**0.909**	**0.88**
	ESM2	0.745	0.779	0.778	0.785	0.58
	ESM3	0.720	0.764	0.762	0.770	0.89
	InterPro	0.865	0.678	0.628	0.629	0.66
	DeepGOZero	0.751	0.677	0.600	0.574	0.21
	DeepGOSE	0.750	0.745	0.672	0.659	0.58

Table S8. (C) Performance comparison under different sequence identity thresholds (continued): Se-quence identity ≤ 50%.

**Sequence identity** ≤ 50%						
**Ontology**	**Method**	**Fmax**	**AUPR**	**ICAUPR**	**DPAUPR**	**thr_fmax**
BPO	IonFusion	**0.727**	**0.621**	**0.573**	**0.566**	**0.76**
	ESM2	0.576	0.581	0.505	0.495	0.63
	ESM3	0.550	0.513	0.446	0.453	0.62
	InterPro	0.577	0.510	0.418	0.413	0.64
	DeepGOZero	0.522	0.435	0.422	0.389	0.40
	DeepGOSE	0.520	0.445	0.350	0.316	0.36
CCO	IonFusion	**0.743**	**0.756**	**0.508**	**0.509**	**0.62**
	ESM2	0.519	0.446	0.449	0.448	0.57
	ESM3	0.456	0.497	0.467	0.462	0.62
	InterPro	0.684	0.574	0.497	0.493	0.61
	DeepGOZero	0.441	0.454	0.280	0.279	0.09
	DeepGOSE	0.724	0.694	0.483	0.452	0.33
MFO	IonFusion	**0.912**	**0.926**	**0.911**	**0.912**	**0.48**
	ESM2	0.747	0.791	0.772	0.773	0.44
	ESM3	0.736	0.708	0.719	0.718	0.36
	InterPro	0.884	0.588	0.529	0.525	0.46
	DeepGOZero	0.737	0.688	0.567	0.549	0.41
	DeepGOSE	0.728	0.753	0.648	0.633	0.37

Table S9. Out-of-vocabulary GO annotations in the test sets. The prediction vocabulary contains unique GO terms observed in the corresponding training split. OOV annotations are test annotation occurrences whose GO terms are absent from that vocabulary. Affected proteins have at least one OOV annotation. Percentages use the total number of propagated test annotation occurrences or test proteins as the denominator. Within each threshold–ontology setting, the vocabulary and OOV set are method-independent; all methods were evaluated against the same propagated ground truth with OOV terms retained in the recall denominators.

Identity	Ontology	Test proteins	Training vocabulary	Test annotations	OOV annotations	OOV ann. (%)	Affected proteins	Affected (%)
10%	BPO	173	2106	8269	613	7.41	58	33.53
10%	CCO	305	415	3462	15	0.43	9	2.95
10%	MFO	313	364	9240	137	1.48	42	13.42
30%	BPO	173	2065	5288	233	4.41	45	26.01
30%	CCO	305	411	3214	22	0.68	12	3.93
30%	MFO	313	429	8189	26	0.32	17	5.43
50%	BPO	173	2295	6182	191	3.09	31	17.92
50%	CCO	305	388	3394	26	0.77	18	5.90
50%	MFO	313	422	7238	24	0.33	13	4.15

Table S10. The six most frequent highest-attention five-residue windows among 7,515 correctly classified potassium-channel proteins. Each protein contributes one five-residue window with the highest attention-derived score, and the first six rows list the most frequent individual sequences ranked by occurrence. In addition, the final row reports the cumulative number and proportion of proteins whose highest-attention window is either exact `TVGYG` or differs from `TVGYG` by one substitution at positions 1–4, with the fifth residue constrained to G; this cumulative count includes exact `TVGYG`. These statistics refer only to the highest-attention window of each protein rather than motif occurrence anywhere in the full sequence.

Frequency rank	Sequence	Proteins (*n*)	Proportion (%)
1	`TVGYG`	1,710	22.75
2	`TIGFG`	738	9.82
3	`TLGYG`	466	6.20
4	`TIGYG`	400	5.32
5	`AAFWY`	379	5.04
6	`CTIGY`	349	4.64
–	`TVGYG`-like	2,975	39.59

Table S11. Occurrence of `TVGYG`-like sequences across the nine fine-grained classes in the final embedding-complete classification test set (n = 24,080; class denominators are reported in Supplementary Table S2). Proteins were grouped according to their true class labels and counted at most once under each criterion, regardless of the number of motif occurrences. The `TVGYG`-like criterion includes exact `TVGYG` and five-residue windows differing at one of positions 1–4; the fifth residue must remain G. This position-restricted rule reflects the strong functional constraint on the terminal glycine observed in KcsA mutagenesis, as cited in the main text. Percentages were calculated relative to the total number of proteins in each fine-grained class. Motif occurrence was assessed across the full-length sequence for all analyzed test proteins, regardless of prediction correctness.

Protein class	Exact `TVGYG`, *n* (%)	`TVGYG`-like, *n* (%)
Ion transporter	0 (0.00)	44 (1.27)
Potassium channel	2,594 (34.13)	5,165 (67.96)
Calcium channel	0 (0.00)	21 (0.59)
Sodium channel	5 (0.06)	71 (0.86)
Chloride channel	0 (0.00)	15 (3.87)
Cys-loop receptor channel	0 (0.00)	0 (0.00)
Ionotropic glutamate channel	0 (0.00)	0 (0.00)
ATP-gated channel	78 (48.45)	124 (77.02)
Proton-gated channel	0 (0.00)	0 (0.00)

Table S12. IonFusion performance on three nested test subsets derived from the original `predict_50` test set for biological process (BPO), cellular component (CCO), and molecular function (MFO). The original dataset was constructed using a 50% CD-HIT sequence-identity threshold. We then used MMseqs2 to calculate the maximum sequence identity of each test protein to the training set and selected proteins with maximum identities below 10% and 30% to form the 10% and 30% subsets, respectively. The 50% group represents the complete original test set.

Ontology	Test subset	Proteins (n)	Fmax	AUPR	ICAUPR	DPAUPR
BPO	$<$ 10% identity	53	0.445	0.450	0.372	0.361
BPO	$<$ 30% identity	66	0.506	0.511	0.442	0.434
BPO	Original test set	173	0.727	0.621	0.573	0.566
CCO	$<$ 10% identity	63	0.517	0.516	0.326	0.324
CCO	$<$ 30% identity	82	0.553	0.557	0.379	0.384
CCO	Original test set	305	0.743	0.756	0.508	0.509
MFO	$<$ 10% identity	38	0.716	0.783	0.619	0.634
MFO	$<$ 30% identity	52	0.793	0.831	0.728	0.739
MFO	Original test set	313	0.912	0.926	0.911	0.912
